# Establishment of a Simple Pediatric Lower Respiratory Tract Infections Database Based on the Structured Electronic Medical Records

**DOI:** 10.3389/fped.2022.917994

**Published:** 2022-06-16

**Authors:** Jilei Lin, Shuhua Yuan, Bin Dong, Jing Zhang, Lei Zhang, Jinhong Wu, Jiande Chen, Mingyu Tang, Bin Zhang, Hansong Wang, Liangye Xu, Liebin Zhao, Yong Yin

**Affiliations:** ^1^Department of Respiratory Medicine, Shanghai Children's Medical Center, School of Medicine, Shanghai Jiao Tong University, Shanghai, China; ^2^Pediatric AI Clinical Application and Research Center, Shanghai Children's Medical Center, Shanghai, China; ^3^Shanghai Engineering Research Center of Intelligence Pediatrics (SERCIP), Shanghai, China; ^4^Department of Information, Shanghai Children's Medical Center, Shanghai Jiao Tong University School of Medicine, Shanghai, China

**Keywords:** children, lower respiratory tract infections, database, structured electronic medical records, hospitalization

## Abstract

**Objective:**

This study aimed to establish a pediatric lower respiratory tract infections (PLRTIs) database based on the structured electronic medical records (SEMRs), to provide a brief overview and the usage process of the SEMRs and the database.

**Methods:**

All the medical information is recorded by a clinical information system developed by Eureka Systems Company. A plugin of the software was used to set the properties of items of the SEMR. Children with lower respiratory tract infections (LRTIs) who were admitted to the department of respiratory medicine of our hospital from May 2020 were included. PostgreSQL 13.1 software was used to construct the PLRTIs database.

**Results:**

Seven kinds of SEMRs were established, and the admission record was the most important and complex among them. It was mainly composed of 10 parts, i.e., basic information, history of present illness, past history (without respiratory disease), past history of respiratory diseases, personal history, family history, physical examination, the score of LRTIs, auxiliary examination, and diagnosis. With the three-level doctor ward round, the recorded information of the SEMR would be checked repeatedly, thus guaranteeing the correctness of the information. The data of the SEMR and laboratory tests could be extracted directly from the hospital information system (HIS) by PostgreSQL 13.1 software with the specific structured query language (SQL) code. After manually checking the original records, the datasets were imported into PostgreSQL 13.1 software, and a simple PLRTIs database was constructed. According to the inclusion criteria of this study, a total of 1,184 children with LRTIs were included in this database from 1 May 2020 to 30 April 2021.

**Conclusion:**

A series of SEMRs for PLRTIs were designed and used during the hospitalization. A simple PLRTIs database was established based on the SEMR. The SEMRs will provide complete and high-quality data on LRTIs in children.

## Introduction

Respiratory diseases are common in children, causing serious health problems worldwide. Lower respiratory tract infections (LRTIs) greatly contribute to the hospitalization of children with an increasing rate risen by 290% from 2000 to 2015. (1) LRTIs account for a substantial part of causes leading to pediatric morbidity and mortality. (2) The characteristics of pediatric lower respiratory tract infections (PLRTIs) vary greatly with different pathogens and severity. Therefore, managing the massive clinical data well will contribute to the development of precision medicine in PLRTIs.

Electronic medical records (EMRs) are widely used in hospitals, and a large amount of clinical data are generated by EMRs in daily medical activities. (3) In recent years, the Surveillance, Epidemiology, and End Results (SEER) and Medical Information Mart for Intensive Care (MIMIC) databases have been known worldwide (4, 5), which provided clinicians with numerous clinical data to deal with. Clinicians and researchers have realized that the clinical data generated by the actual clinical practices in their own centers may help to provide more appropriate solutions for the medical problems in their daily work. Therefore, EMRs will play an important role in improving medical services. However, the inconsistency of format of records, missing values, and the difficulty of data extraction from the text format became the main causes of poor utilization of the clinical data. How to use the clinical data efficiently and accurately has emerged to become a problem that urgently needs to be settled.

Considering the factors above, we tried to establish a structured electronic medical record (SEMR) of PLRTIs to unify the format of the medical records and construct a simple PLRTIs database based on the SEMR to facilitate data extraction and enable the mining of clinical data. Furthermore, the standardized processes for maintaining the operation of this database designed by us were also introduced in this study.

## Materials and Methods

### Data Source

This study was conducted in Shanghai Children's Medical Center, a 1,000-bed tertiary teaching hospital in Shanghai, China. Patients who were hospitalized for LRTIs in the department of respiratory medicine of Shanghai Children's Medical Center were enrolled starting from 1 May 2020. The study was approved by the Ethics Committee of Shanghai Children's Medical Center and conducted according to the Declaration of Helsinki guidelines. The information of all patients was handled anonymously.

### Study Population

The eligibility criteria were as follows: (1) hospitalized children, (2) aged between 1 month and 18 years old, and (3) diagnosed with LRTIs (see definitions).

The exclusion criteria were any of the following: (1) the guardians refused to sign the obtaining informed consent, (2) the guardians asked for automatically discharging, or (3) information was seriously absent.

### Definitions of LRTIs

Lower respiratory tract infections s were diagnosed mainly based on the clinical manifestations and pulmonary physical examination. The diagnosis may be revised by the chest imaging results during hospitalization. The main clinical symptoms are cough, wheeze, and fever. The main diseases of LRTIs include pneumonia, bronchitis, bronchiolitis, and etc. After the resident doctors identify the children with LRTIs on the first day of admission, all the diagnoses (specific types of LRTIs) will be confirmed with at least two different senior attending physicians or chief physicians during hospitalization.

### Establishment of SEMR

In our hospital, all the medical information is recorded in a clinical information system that is developed by Eureka Systems Company. A plugin of the medical record software was used to set the properties of items of the SEMR. The SEMR is a structured template of medical records we made in our old medical record system (HIS) that can be filled in by doctors, mostly in the form of multiple choice and with pre-specified contents. The name of every item was used to remind clinicians what contents need to be filled in. For numerical data, we limited their ranges and units. Finally, seven kinds of SEMRs were established based on the EMR system we currently use, such as admission records, attending physician's ward round record on the first day of admission, the chief physician's ward round record on the second day of admission, daily course of diseases, etiological assessments, security events during hospitalization, and adverse effects of medicines. The data generated with the SEMRs were extracted by our teams from HIS with the permission of the hospital.

### Data Storage and Extraction

Clinicians recorded the medical records by using the original software (HIS), but the contents of the records were pre-specified with SEMR. The clinical data from the SEMR were stored in HIS in proprietary formats. All items of the SEMR provided routines that could import/export Extensible Markup Language (XML) data with a known XML schema in HIS. Because the PostgreSQL software has good support for data in the XML or JSON format, a structured query language (SQL) script can be generated to extract all data from the HIS, and export them in Excel format by PostgreSQL 13.1. (6) Each object has a unique ID to associate it with all relevant information. The data of the SEMR were extracted by our teams from HIS to import into the PLRTIs database with the permission of the hospital, and all extracted data should be anonymous.

### Chinese Characters and Data Management

Since many Chinese characters existed in the datasets, we imported these data into R 4.10 software to convert them into numerical values for post-processing. (7) Outliers and missing data were found by R 4.10 software, and the running codes in R 4.10 software were written to check the interrelated data and general data. All abnormal information was corrected by manually checking the original records.

## Results

### Design of the SEMR

**Figure 1:**
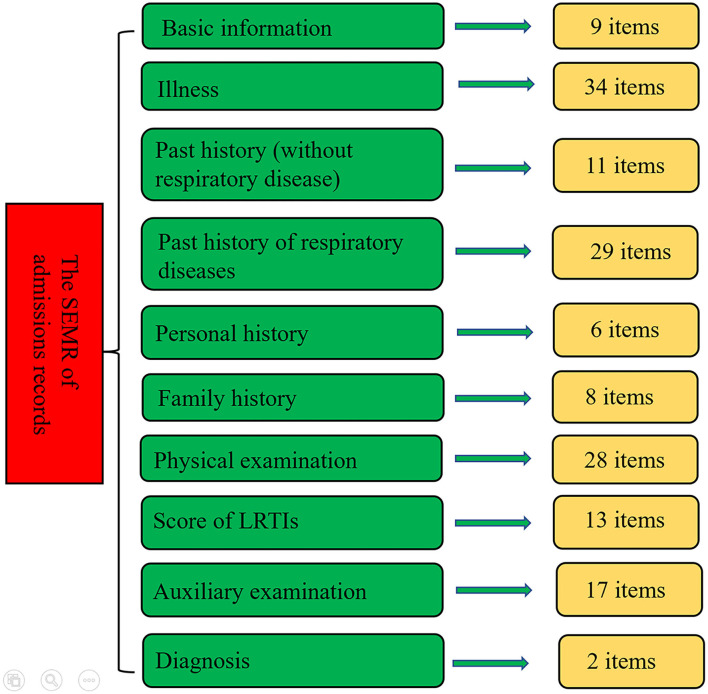
The outline of the structured electronic medical record (SEMR) of admission record.

Due to the lack of standardized terminology for PLRTIs, we identified a series of medical terms through discussions with several experts of pediatric respiratory diseases. The admission record was the most important and complex part. It was mainly composed of 10 parts, i.e., basic information, history of present illness, past history (without respiratory disease), past history of respiratory diseases, personal history, family history, physical examination, the score of LRTIs, the auxiliary examination, and diagnosis. In the part of the history of present illness, the following common information was recorded: pre-specified symptoms (such as fever, cough, wheeze, and etc.), duration, severity, and frequency. For most items, resident doctors only need to choose the corresponding options. The outline of the SEMR for the admission records is shown in Figure 1. The detailed information is shown in Supplementary Table S1. Complete templates of all of the SEMRs are available from the corresponding author upon reasonable request.

### Process and Standards for Usage of the SEMR

**Figure 2:**
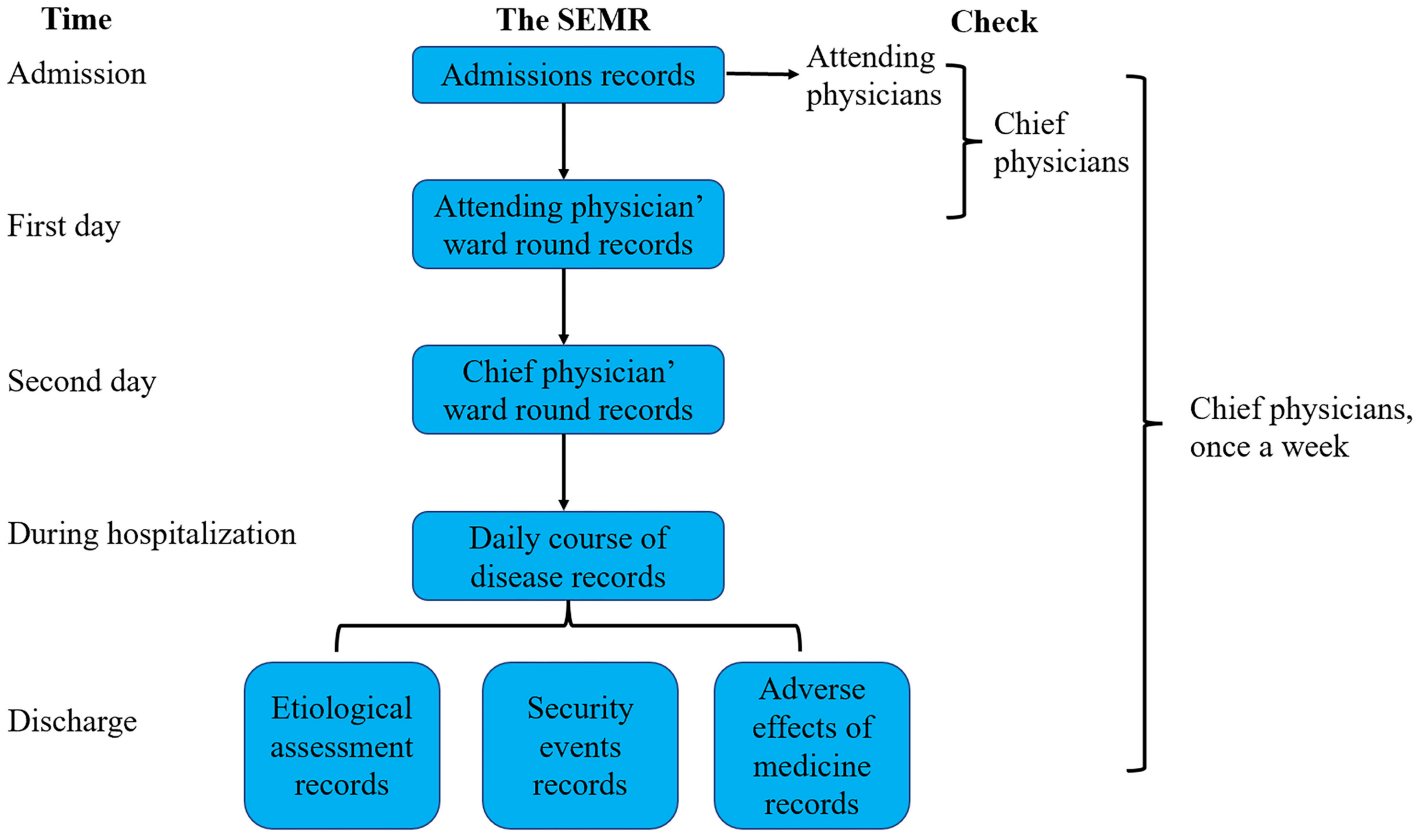
The flow chart of the process of usage of the structured electronic medical record.

First of all, all physicians who recorded the information of patients would be trained about the meanings of every item. From 1 May 2020, the SEMR was used for all children who were admitted to the department of respiratory disease. Detailed information of each patient was inquired and recorded in SEMR by the trained physicians. On the first day of hospitalization, attending physicians would check the correctness of the admission records, and any errors in the SEMR of the admission record would be corrected immediately. In addition, the SEMR of attending physician's ward round records would be completed by resident physicians with the guidance of attending physicians. On the second day of hospitalization, chief physicians would check the correctness of the admission record and attending physician's ward round records. In addition, the SEMR of the chief physician's ward round records would be completed by resident physicians with the guidance of chief physicians. When the patients were discharged from the hospital, physicians needed to complete etiological assessments, security events during hospitalization, and adverse effects of medical records. The chief physician will check all the medical records of inpatients and recently discharged patients once a week. The flow chart of the process of usage of the SEMR is shown in Figure 2.

### Data Collection and Extraction

The number of admissions in the department of respiratory disease was 2,079 till 30 April 2021. Because it is not a clinical intervention study, all the processes of our diagnosing and treatment are as usual, and the choice of therapies for each patient has not been interfered. Some routine and common laboratory tests of blood and sputum were conducted on these patients, the detailed contents of the tests are shown in Table 1. The examination items were adjusted according to the children's symptoms.

**Table 1:** The package of auxiliary examinations for children with lower respiratory tract infections (LRTIs).

**Sample**	**Items**
**Blood**	Blood routine+ C-reactive protein+ Serum Amyloid A Protein
	Hepatic and renal function+ Cardiac troponin I
	Electrolyte+ Lactic dehydrogenase
	Creatine kinase+ Creatine Kinase Isoenzymes MB
	Coagulation Function
	Procalcitonin
	Interleukin-6
	Ferroprotein
	Erythrocyte sedimentation rate
	Vitamin D
	Blood culture (only children with fever)
	Peripheral blood monocyte activation assay
	Absolute value and relative proportion of T cell subsets, B cells and NK cells
	Regulatory T cell
	Immunoglobulin(IgA, IgG, IgM)
	Total IgE + 29 kinds of specific allergen IgE absolute value
**Sputum**	7 kinds of respiratory pathogens
	Mycoplasma pneumoniae RNA
	Mycoplasma pneumoniae and Chlamydia pneumoniae DNA
	Human rhinovirus RNA
	Mycoplasma Pneumoniae Antibodies
	Legionella pneumophila DNA
	Group A Streptococcus antigen
	Drug-resistance gene of mycoplasma pneumoniae+ bordetella pertussis
	Sputum bacterial culture
**Urine**	Routine urine test
	Streptococcus pneumoniae antigen
**Feces**	Fecal routine

With the SEMR, the format and quantity of each patient's medical data stored in HIS were similar. The XML data of each patient in HIS could be imported/exported easily with a routine (Supplementary File S1). The medical data of the SEMR, laboratory tests, and therapies could be extracted directly in batches from the HIS by PostgreSQL 13.1 software with specific SQL code (a part of the codes is shown in Supplementary File S2).

### The Simple Database Construction

**Figure 3:**
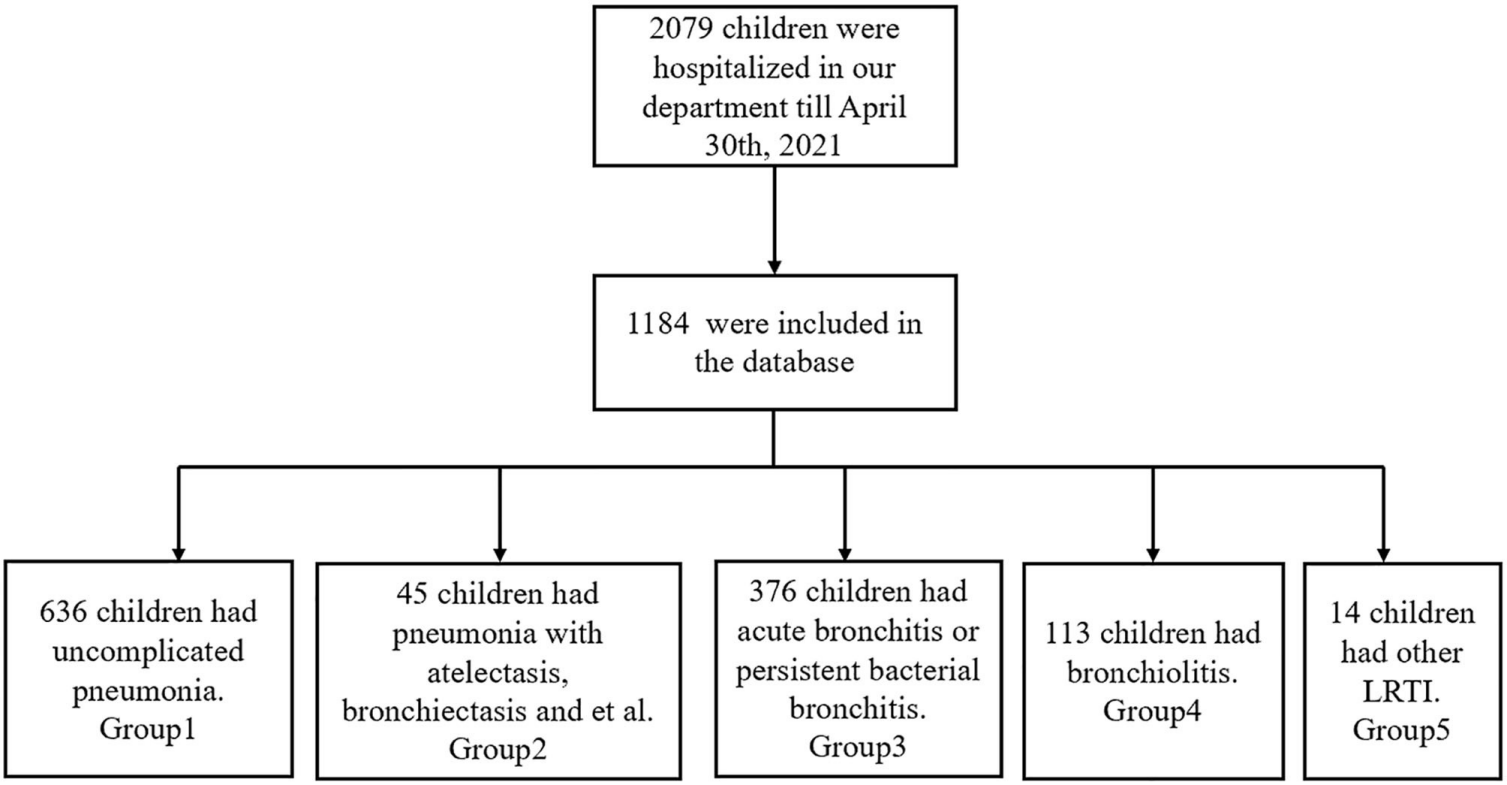
Flow diagram of the selected cases.

According to the inclusion criteria of this study, a total of 1,184 children with LRTIs were included in all structured EMR from 1 May 2020 to 30 April 2021. The flow diagram of inclusion is shown in Figure 3. Clinical medical records, laboratory examinations, and therapies were stored in 3 Excel files, respectively. The “CureNo” was a unique identification code in 3 files to associate corresponding items. Because there are many Chinese characters in the datasets, we imported these data into R software to convert them into numerical values for the post-processing of data. The highest proportion of missing values of an attribute of all entities was only 2.45%. We collated the abnormal and missing values and tried to fill or correct these values by manually checking the original records. Similar to the MIMIC database, the SQL codes were designed to import these datasets into PostgreSQL 13.1 software, and a simple PLRTIs database was constructed. The detailed information of the clinical data in the database is shown in Supplementary Table S1.

**Figure 4:**
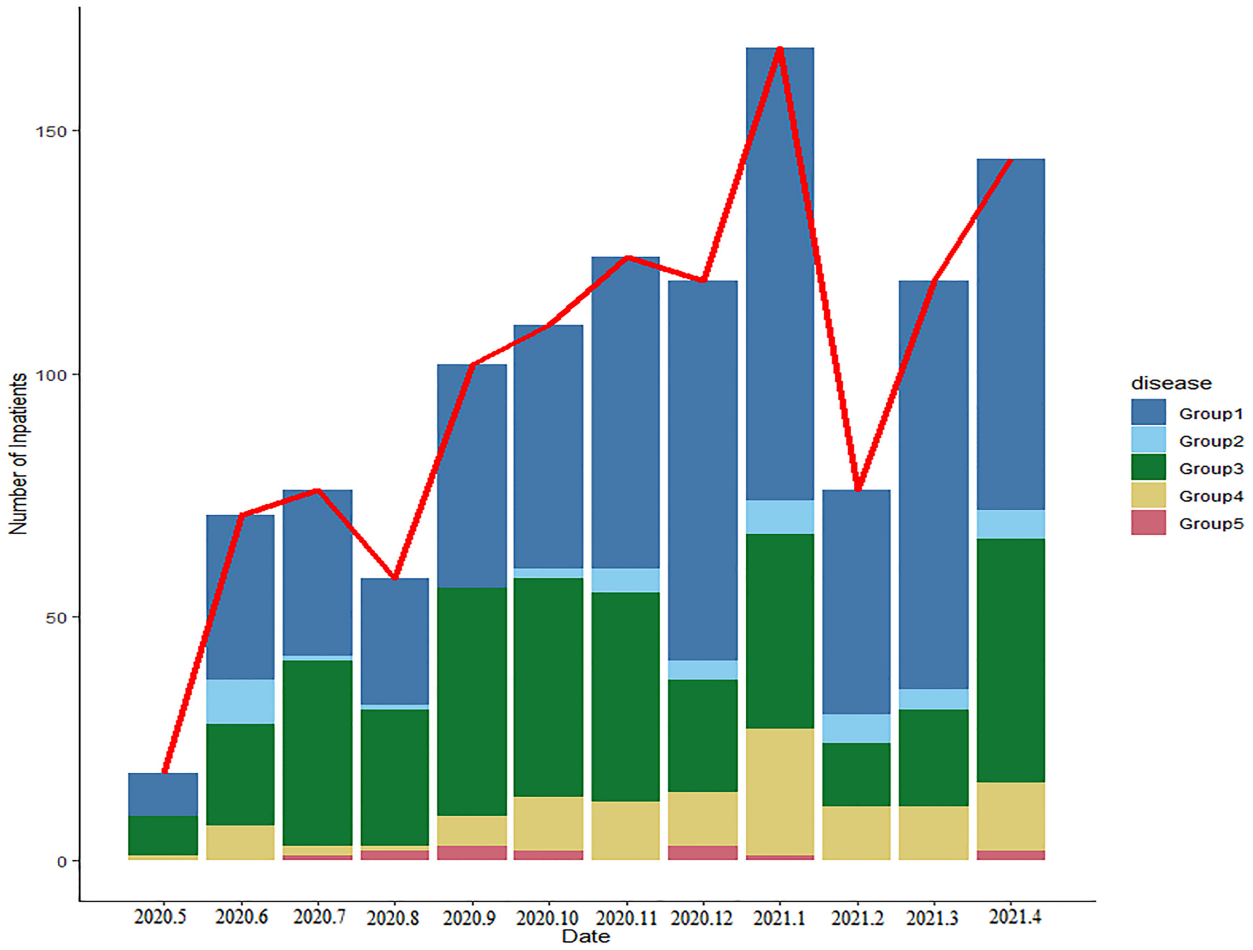
The changes in the number and proportions of children with lower respiratory tract infections (LRTIs) per month in this database.

In order to clearly classify the subtype of LRTIs, we have divided these children into 4 groups in the PLRTIs database according to their main diagnosis. The diagnostic information of all patients included Chinese text and ICD10 code, which were analyzed by R software. In total, six hundred and thirty-six children with uncomplicated pneumonia were included in Group 1. Totally, forty-five children with pneumonia complicated with atelectasis or bronchiectasis, were included in Group 2. In total, three hundred and seventy-six children with acute bronchitis and persistent bacterial bronchitis were included in Group 3 and one hundred and thirteen children with bronchiolitis were included in Group 4. In total, fourteen children with laryngotracheobronchitis, chronic bronchitis, and tuberculosis were included in Group 5. In order to understand the impact of the COVID-19 pandemic on LRTIs of our hospitalized patients, we plotted a figure of patients admitted between May 2020 and April 2021 (Figure 4).

## Discussion

A series of SEMRs for PLRTIs were designed and used during hospitalization, and the information of patients was recorded completely in a uniform format. The simple PLRTIs database was established based on the SEMR. Currently, the database is constantly generating clinical data with good completeness in a fixed format. This study provided a brief overview of the SEMRs database, and the process and standards for the usage of the SEMRs.

Lower respiratory tract infection is the main cause of hospitalization for children, and many studies were conducted on it. (8, 9) However, there are many problems that have not been well answered, such as early diagnosis, choice of treatment, and their outcomes. (10) In recent years, artificial intelligence (AI) has been widely used in the clinical medicine, many studies have established predictive models for the diagnosis and treatment using AI methods. (11) Song et al. used machine learning to identify patients with non-small cell lung cancer who would not benefit from epidermal growth factor receptor tyrosine kinase inhibitor therapy (EGFR-TKI therapy) (12), and the results provided the important guidelines for clinical practices in patients with non-small cell lung cancer. As we know, a good utilization of AI clinical application is based on massive data, and the high-quality data are the most important elements. Therefore, we must ensure that the data generated from the clinical practice are correct, complete, and easy to get, so that they can provide the basis for AI and help to improve traditional clinical decision-making. Therefore, this database provides high-quality data, which may promote the application of AI in children's respiratory diseases in the future.

Although every physician was trained before filling these items, and senior attending physicians and chief physicians had checked these records every day, there were still some missing values and outliers. In this study, we found the main reason for the missing value is that some physicians deleted some items at beginning because they thought the information was not important according to their own habits. As for outliers, some physicians have changed the unit of values (such as, from g to kg in weight), and sometimes Chinese characters are used in the numeric items. Therefore, we had adopted several methods to solve these problems: firstly, most of the items were reset so that they cannot be deleted; secondly, we made range and unit settings for each numeric item; thirdly, we extracted the data from the database every 3 months, and designated a physician to sort out the wrong or missing data and manually correct them.

We randomly chose several admission records recorded with or without the SEMR and compared these 2 kinds of admission records. Interestingly, the time for finishing the SEMR of admission records did not increase significantly when compared with the normal EMR. This result was not presented in the study. This finding suggested that although more information was recorded with the SEMR, it did not significantly increase the work intensity of physicians in clinical practices. The reason may be that most items in SEMR were choice questions with pre-specific answers, which helped physicians to be clear about the clinical information they needed to collect and saved the time of writing medical records.

There are some deficiencies in the HIS system we currently use. First of all, clinicians are not familiar with the data extraction process of the HIS, and the data storage structure is complex for most clinicians. When you want to extract the data, you need the help of engineers in the medical records department. Secondly, data extraction must be performed with the specific internet of the hospital, of which the running speed is too slow to complete the task. Finally, all patients' information is unmasked in HIS, which may lead to information leakage, besides, it is possible to modify the original data in the process of data extraction with unmasked information. On the contrary, the patients' information was handled anonymously in our database, and the data extraction in our database can be performed without the internet. However, we must emphasize that we do not want to replace HIS with this simple database. We hope to share our experience with other clinicians on how to collect better clinical data.

The strengths of this study are highlighted. To our knowledge, this is the first study to establish a PLRTIs database. This database will provide a large number of clinical data with high quality for researchers. Secondly, the SEMRs provide convenience for data extraction and sorting. Furthermore, this study described the multiple inspection processes of data collection in clinical practices, which is conducive to improve the quality of data and is worth promoting.

The weak points of this study are mainly that there are still some missing values and outliers, though we have proposed solutions above. In the future, we plan to develop an AI assistant to remind doctors with the potential mistakes in time and to send messages to the database managers. Secondly, although we can roughly process Chinese characters with R software, how to deal with Chinese characters is a thorny problem since it is difficult to extract valuable information from a long sentence. In the future, we need to develop natural language processing techniques to deal with these complex situations.

This study is a preliminary study on how to collect more clinical information and reduce the waste of human resources in extracting the data from the different text records with Chinese characters. If allowed, all original data without identified information of patients and relevant materials of this database may be uploaded to the internet. In addition, our next research will mainly introduce the detailed information about these datasets and how to use these data. Therefore, clinicians of pediatric respiratory diseases will have the opportunity to use these data with appropriate requests to solve clinical problems.

## Conclusion

A series of SEMR for PLRTIs were designed and used during hospitalization. A simple PLRTIs database was established based on SEMR. We have developed the process of using SEMR, as well as the processes and standards of data acquisition and supervision. The SEMR will provide complete and high-quality data on LRTIs in children, and it may help to provide the evidence for the unsettled issues.

## Data Availability Statement

The original contributions presented in the study are included in the article/Supplementary Material, further inquiries can be directed to the corresponding author.

## Ethics Statement

The studies involving human participants were reviewed and approved by the Ethics Committee of Shanghai Children's Medical Center, Shanghai Jiao Tong University School of Medicine and conducted according to the Declaration of Helsinki guidelines. Written informed consent to participate in this study was provided by the participants' legal guardian/next of kin.

## Author Contributions

JL, SY, and BD conceptualized and designed the study, supervised data collection, carried out the initial analyses, and drafted the initial manuscript. JZ, LZhan, and JW designed the data collection instruments and collected data. JC, MT, BZ, and HW coordinated and supervised data collection, assisted in the statistical analysis, and carried out the initial analyses. YY, LZhao, and LX conceptualized and designed the study, supervised data collection, reviewed, and revised the manuscript. All authors read and approved the final manuscript.

## Funding

This work was supported by the Science and Technology Innovation-Biomedical Supporting Program of Shanghai Science and Technology Committee [no. 19441904400]; the Science and Technology Innovation-Biomedical Supporting Program of Shanghai Science and Technology Committee [no. 19441909000]; Program for artificial intelligence innovation and development of Shanghai Municipal Commission of Economy and Informatization [2020-RGZN-02048].

## Conflict of Interest

The authors declare that the research was conducted in the absence of any commercial or financial relationships that could be construed as a potential conflict of interest.

## Publisher's Note

All claims expressed in this article are solely those of the authors and do not necessarily represent those of their affiliated organizations, or those of the publisher, the editors and the reviewers. Any product that may be evaluated in this article, or claim that may be made by its manufacturer, is not guaranteed or endorsed by the publisher.

## Supplementary Material

Supplementary File 1 The Extensible Markup Language (XML) file of the patients in hospital information system (HIS).

Supplementary File 2 The SQL code for extracting the main items of the structured electronic medical record (SEMR).
