# Supplementary material for: Establishment of a Simple Pediatric Lower Respiratory Tract Infections Database Based on the Structured Electronic Medical Records
Source: Front Pediatr. 2022 Jun 16;10:917994. doi: 10.3389/fped.2022.917994 (PMC9243234; doi:10.3389/fped.2022.917994)
Supplement: Supplementary File 1 — The Extensible Markup Language (XML) file of the patients in hospital information system (HIS). [file Data_Sheet_1.PDF]

Table S1: the detailed items of the SEMR and database schema

Topic in SEMR	Name	type
	PatientID	int(11)
	CureNo	int(11)
	BedCode	int(11)
	PatientName	varchar(64)
Basic information	Birthday	datetime
	Age	float
	Sex	varchar(64)
	Admission time	datetime
	Discharge time	datetime
	Chief complaint	text
	History of present illness	text
	Fever (yes/no)	varchar(64)
	Duration of fever	float
	Unit of duration	varchar(64)
Illness	Highest temperatures	float
	Cough (yes/no)	varchar(64)
	Characteristics of cough	varchar(256)
	Cough severity	varchar(256)
	Duration of cough	float
	Unit of duration	varchar(64)

Expectoration (yes/no)	varchar(64)
Wheeze (yes/no)	varchar(64)
Characteristics of cough	varchar(256)
Duration of wheeze	float
Unit of duration	varchar(64)
Foreign body inhalation (yes/no)	varchar(64)
Respiratory concomitant symptoms	varchar(256)
Mental condition (yes/no)	varchar(64)
Characteristics of mental condition	varchar(256)
Appetite (yes/no)	varchar(64)
Characteristics of appetite	varchar(256)
Other systemic symptoms (yes/no)	varchar(64)
Characteristics of other systemic symptoms	varchar(256)
Head and face symptoms (yes/no)	varchar(64)
Characteristics of head and face symptoms	varchar(256)
Upper airway symptoms (yes/no)	varchar(64)
Characteristics of upper airway symptoms	varchar(256)
Skin symptoms (yes/no)	varchar(64)
Characteristics of skin symptoms	varchar(256)
Neurological symptoms (yes/no)	varchar(64)
Characteristics of neurological symptoms	varchar(256)

	Urinary symptoms(yes/no)	varchar(64)
	Characteristics of urinary symptoms	varchar(256)
	Infectious diseases history (yes/no)	varchar(64)
	Which infectious diseases	varchar(256)
	Tuberculosis exposure history(yes/no)	varchar(64)
	Planned immunization (yes/no)	varchar(64)
Past history (without respiratory disease)	Streptococcus pneumoniae vaccination (yes/no)	varchar(64)
	Drug and food allergy (yes/no)	varchar(64)
	Eczema history (yes/no)	varchar(64)
	Time of onset of eczema	varchar(256)
	Milk protein allergy (yes/no)	varchar(64)
	Time of onset of milk protein allergy	varchar(64)
	Allergic rhinitis (yes/no)	varchar(64)
	Chronic respiratory diseases (yes/no)	varchar(64)
Past history of respiratory diseases	Recurrent respiratory tract infection (yes/no)	varchar(64)
	Number of upper respiratory tract infections in past year	int(11)
	Number of lower respiratory tract infections in past year	int(11)

Hospitalization for respiratory diseases (yes/no)	varchar(64)
Times of hospitalization	int(11)
Times of oxygen therapy	int(11)
Times of ICU stay	int(11)
Other respiratory disease (yes/no)	varchar(64)
Severe respiratory diseases (yes/no)	varchar(64)
Other severe disease (yes/no)	varchar(64)
Other respiratory disease (yes/no)	varchar(64)
Wheeze history (yes/no)	varchar(64)
First wheezing (months old)	int(11)
Number of wheezes in 2 years after birth	int(11)
Number of wheezes in 1 years after birth	int(11)
Number of wheezes	int(11)
Precipitating factor for wheeze	varchar(256)
Allergen induced wheezing (yes/no)	varchar(64)
Therapeutic effect of diastolic agent for wheeze	varchar(64)
Test of serum IgE (yes/no)	varchar(64)
Value of total serum IgE	float
Skin prink test (yes/no)	varchar(64)
Results of skin prink test	varchar(256)

	Diagnosed asthma by doctors (yes/no)	varchar(64)
	Start date of asthma treatment	datetime
	Asthma medication	varchar(256)
	Medication compliance	varchar(64)
	Asthma control	varchar(64)
Personal history	Birth weight	float
	Mode of delivery	varchar(64)
	Gestation	float
	Feeding mode	varchar(64)
	Add complementary food	varchar(256)
	Growth and development	varchar(256)
Family history	Father's age	float
	Father's occupation	varchar(256)
	Maternal age	float
	Maternal occupation	varchar(256)
	Father's health condition	varchar(64)
	Mother's health condition	varchar(64)
	Family menber's health condition	varchar(64)
	Smoking exposure (yes/no)	varchar(64)
Physical examination	Temperature measurement method	varchar(64)
	Temperature value	float
	Pulse rate	int(11)

Breath rate	int(11)
Systolic pressure	int(11)
Diastolic pressure	int(11)
Height	float
Weight	float
Oxygen saturation without oxygen therapy	int(11)
Oxygen therapy method	varchar(64)
Oxygen saturation with oxygen therapy	int(11)
Consciousness	varchar(64)
Mental condition	varchar(64)
Nutritional status	varchar(64)
Complexion	varchar(64)
Superficial lymph node	varchar(64)
Thoracic deformity (yes/no)	varchar(64)
Breath status	varchar(64)
Dyspnea (yes/no)	varchar(64)
Tachypnea (yes/no)	varchar(64)
Inspiratory depression (yes/no)	varchar(64)
Symmetrical breath sound	varchar(64)
Crackles (yes/no)	varchar(64)
Position of crackles	varchar(64)
Kinds of crackles	varchar(64)

	Dry rale (yes/no)	varchar(64)
	Position of dry rale	varchar(64)
	Kinds of dry rale	varchar(64)
	Highest temperature in the past 24 hours	float
	Current temperature	float
	Pulse rate	int(11)
	Breath rate	int(11)
	Oxygen saturation	int(11)
	Level of temperature	int(11)
Score of LRTIs	Severity of cough	int(11)
	Severity of expectoration	int(11)
	Severity of wheeze	int(11)
	Severity of chest discomfort during cough	int(11)
	Severity of dyspnea	int(11)
	Pulmonary auscultation	int(11)
	Sum of these scores	int(11)
	COVID-19 nucleic acid detection (yes/no)	varchar(64)
	Data of the nucleic acid detection	varchar(64)
Auxiliary examination	Positive result of COVID-19 test (yes/no)	varchar(64)
	Routine blood test and radiological examination within 3 days	text

	Routine blood test and radiological examination within 7 days	text
	Other tests	text
	Chest X-ray test before hospitalization (yes/no)	varchar(64)
	Data of the X-ray	datetime
	Exudation condition	int(11)
	Position of the infections	int(11)
	Pleural effusion	int(11)
	Abscess, lung cyst or bulla	int(11)
	Atelectasis	int(11)
	Chest X-ray score	int(11)
	Chest CT before hospitalization (yes/no)	varchar(64)
	Data of the CT	datetime
	Positive result of CT (yes/no)	varchar(64)
Diagnosis	Diagnosis	text
	Type of diagnosis	int(11)

Supplementary file1: the XML file of the patients in HIS

<DocObjContent><Section Id="Section_130105134722176" Type="1" Reserve2=""><NewCtrl Id="数据元 1" Type="3" Reserve2=""><Content_Text>上海交通大学医学院附属上海儿童医学中心 </Content_Text></NewCtrl><NewCtrl Id="数据元 2" Type="3" Reserve2=""><Content_Text>住 院 病 史 录</Content_Text></NewCtrl><NewCtrl Id="Reference_130105134214" Type="3" Reserve2=""><Content_Text>***</Content_Text></NewCtrl><NewCtrl Id="Reference_130105134222" Type="3" Reserve2=""><Content_Text>呼 吸 病 区</Content_Text></NewCtrl><NewCtrl Id="Reference_130105134225" Type="3" Reserve2=""><Content_Text>14</Content_Text></NewCtrl><NewCtrl Id="Reference_130408094830" Type="3" Reserve2=""><Content_Text>64626659</Content_Text></NewCtrl><NewCtrl Id="Reference_130105134228" Type="3" Reserve2=""><Content_Text>413636</Content_Text></NewCtrl><Content_Text>
上海交通大学医学院附属上海儿童医学中心 第 页
住 院 病 史 录
姓名: ***
病区: 呼吸病区
床位号: 14
门诊号: ****
住院号: ****
</Content_Text></Section><Section Id="InhospitalRecord" Type="1"

Reserve2=""><Content_Text>入院记录</Content_Text></Section><Section Id="Section_130105135123267" Type="1" Reserve2=""><NewCtrl Id="数据元6" Type="3" Reserve2=""><Content_Text>姓名：</Content_Text></NewCtrl><NewCtrl Id="Reference_130105120200" Type="3" Reserve2=""><Content_Text>****</Content_Text></NewCtrl><NewCtrl Id="数据元54" Type="3" Reserve2=""><Content_Text>家长姓名：</Content_Text></NewCtrl><NewCtrl Id="Reference_****" Type="3" Reserve2=""><Content_Text>****</Content_Text></NewCtrl><NewCtrl Id="数据元17" Type="3" Reserve2=""><Content_Text>性别：</Content_Text></NewCtrl><NewCtrl Id="Reference_****" Type="3" Reserve2=""><Content_Text>男</Content_Text></NewCtrl><NewCtrl Id="数据元56" Type="3" Reserve2=""><Content_Text>联系电话：</Content_Text></NewCtrl><NewCtrl Id="Reference_*" Type="3" Reserve2=""><Content_Text>**</Content_Text></NewCtrl><NewCtrl Id="数据元25" Type="3" Reserve2=""><Content_Text>年龄：</Content_Text></NewCtrl><NewCtrl Id="Reference_*" Type="3" Reserve2=""><Content_Text>****</Content_Text></NewCtrl><NewCtrl Id="数据元57" Type="3" Reserve2=""><Content_Text>户口地址：</Content_Text></NewCtrl><NewCtrl Id="Reference_130407102118" Type="3" Reserve2=""><Content_Text>****294</Content_Text></NewCtrl><NewCtrl Id="数据元26" Type="3" Reserve2=""><Content_Text>出生日期：</Content_Text></NewCtrl><NewCtrl Id="Reference_130407165626" Type="3"

Reserve2=""><Content_Text>****</Content_Text></NewCtrl><NewCtrl Id="数据元 58" Type="3" Reserve2=""><Content_Text>现住址：</Content_Text></NewCtrl><NewCtrl Id="Reference_130407165616" Type="3" Reserve2=""><Content_Text>****294</Content_Text></NewCtrl><NewCtrl Id="数据元 27" Type="3" Reserve2=""><Content_Text>出生地：</Content_Text></NewCtrl><NewCtrl Id="Reference_130403131419" Type="3" Reserve2=""><Content_Text>****</Content_Text></NewCtrl><NewCtrl Id="数据元 59" Type="3" Reserve2=""><Content_Text>入院日期：</Content_Text></NewCtrl><NewCtrl Id="Reference_****" Type="3" Reserve2=""><Content_Text>2020-05-1211:05</Content_Text></NewCtrl><NewCtrl Id="数据元 33" Type="3" Reserve2=""><Content_Text>民族：</Content_Text></NewCtrl><NewCtrl Id="Reference_130105120239" Type="3" Reserve2=""><Content_Text>汉族</Content_Text></NewCtrl><NewCtrl Id="数据元 61" Type="3" Reserve2=""><Content_Text>记录日期：</Content_Text></NewCtrl><NewCtrl Id="记录日期" Type="11" Reserve2=""><Content_Text>2020-05-12 13:17</Content_Text></NewCtrl><NewCtrl Id="数据元 44" Type="3" Reserve2=""><Content_Text>供史者：</Content_Text></NewCtrl><NewCtrl Id="数据元 62" Type="3" Reserve2=""><Content_Text>门急诊入院诊断：</Content_Text></NewCtrl><NewCtrl Id="Reference_130105120304" Type="3" Reserve2=""><Content_Text>社区获得性肺炎非重症；败血症其他特指</Content_Text></NewCtrl><NewCtrl Id="数据元 45" Type="3" Reserve2=""><Content_Text>诊断日期：</Content_Text></NewCtrl><NewCtrl Id="诊断日

期 " Type="11" Reserve2=""><Content_Text>2020-05-12 13:17</Content_Text></NewCtrl><Content_Text>姓名：****

家长姓名：****

性别：男

联系电话：****

年龄：****

户口地址：****

出生日期：****

现住址：****

出生地：****

入院日期：****

民族：****

记录日期：****

供史者：患儿家属

门急诊入院诊断：****

诊断日期：2020-05-12 13:17

</Content_Text></Section><NewCtrl Id=" 数 据 元 64" Type="3" Reserve2=""><Content_Text>48 小 时 内 主 治 医 师 诊 断 ： </Content_Text></NewCtrl><Section Id="section130204100111" Type="1" Reserve2=""><Section Id="section130204100141" Type="1" Reserve2=""><NewCtrl Id="数 据 元 3" Type="3" Reserve2=""><Content_Text /></NewCtrl><Content_Text /></Section><Content_Text /></Section><NewCtrl Id=" 数 据 元 66" Type="3"

Reserve2=""><Content_Text>补充诊断：</Content_Text></NewCtrl><NewCtrl Id="数据元 32" Type="3" Reserve2=""><Content_Text /></NewCtrl><Section Id="主诉" Type="1" Reserve2=""><NewCtrl Id="数据元 67" Type="3" Reserve2=""><Content_Text>1.主诉：</Content_Text></NewCtrl><Section Id="Section_ChiefComplaint" Type="1" Reserve2=""><Content_Text>**** </Content_Text></Section><Content_Text>1.主诉：**** </Content_Text></Section><NewCtrl Id="数据元 68" Type="3" Reserve2=""><Content_Text>2.现病史：</Content_Text></NewCtrl><Section Id="科室现病史" Type="1" Reserve2=""><Content_Text>**** </Content_Text></Section><NewCtrl Id="有无发热" Type="7" Reserve2=""><Content_Text>有 </Content_Text></NewCtrl><NewCtrl Id="数据元 15" Type="3" Reserve2=""><Content_Text>11</Content_Text></NewCtrl><NewCtrl Id="时长" Type="7" Reserve2=""><Content_Text>天</Content_Text></NewCtrl><NewCtrl Id="体温测量方式 1" Type="7" Reserve2=""><Content_Text>耳温</Content_Text></NewCtrl><NewCtrl Id="热峰" Type="3" Reserve2=""><Content_Text>38.7</Content_Text></NewCtrl><NewCtrl Id="发热伴随症状" Type="10" Reserve2=""><Content_Text>无 </Content_Text></NewCtrl><NewCtrl Id="咳嗽时相" Type="10" Reserve2=""><Content_Text>日夜均咳</Content_Text></NewCtrl><NewCtrl Id="咳嗽天数" Type="5" Reserve2=""><Content_Text>160</Content_Text></NewCtrl><NewCtrl Id="是否咳痰" Type="10" Reserve2=""><Content_Text>湿性咳嗽（有痰咳嗽） </Content_Text></NewCtrl><NewCtrl Id="咳嗽性质" Type="10" Reserve2=""><Content_Text>咳剧时面色涨红</Content_Text></NewCtrl><NewCtrl Id="咳嗽严重程度" Type="10" Reserve2=""><Content_Text>频繁咳嗽，不影响日常活动

</Content_Text></NewCtrl><NewCtrl Id="喘息持续时间" Type="10" Reserve2=""><Content_Text>无</Content_Text></NewCtrl><NewCtrl Id="喘息天数" Type="5" Reserve2=""><Content_Text>0</Content_Text></NewCtrl><NewCtrl Id="时长2" Type="7" Reserve2=""><Content_Text>天</Content_Text></NewCtrl><NewCtrl Id="喘息伴随症状" Type="10" Reserve2=""><Content_Text>无伴随症状</Content_Text></NewCtrl><NewCtrl Id="异物吸入" Type="7" Reserve2=""><Content_Text>否认</Content_Text></NewCtrl><NewCtrl Id="呼吸情况" Type="10" Reserve2=""><Content_Text>胸壁吸气凹陷</Content_Text></NewCtrl><NewCtrl Id="呼吸伴随症状" Type="10" Reserve2=""><Content_Text>无</Content_Text></NewCtrl><NewCtrl Id="精神情况" Type="10" Reserve2=""><Content_Text>精神正常</Content_Text></NewCtrl><NewCtrl Id="胃纳" Type="10" Reserve2=""><Content_Text>胃纳正常</Content_Text></NewCtrl><NewCtrl Id="消化" Type="10" Reserve2=""><Content_Text>两便正常</Content_Text></NewCtrl><NewCtrl Id="数据元81" Type="3" Reserve2=""><Content_Text>3.既往史：</Content_Text></NewCtrl><Section Id="既往史" Type="1" Reserve2=""><Section Id="传染史" Type="1" Reserve2=""><NewCtrl Id="数据元90" Type="3" Reserve2=""><Content_Text>传染病史：</Content_Text></NewCtrl><NewCtrl Id="是否" Type="7" Reserve2=""><Content_Text>否认</Content_Text></NewCtrl><NewCtrl Id="病症" Type="10" Reserve2=""><Content_Text>麻疹,水痘,猩红热,流行性腮腺炎,肝炎,结核</Content_Text></NewCtrl><Content_Text>传染病史：否认麻疹，水痘，猩红热，流行性腮腺炎，肝炎，结核等病史；</Content_Text></Section><NewCtrl Id="结核接触史" Type="8"

Reserve2=""><Content_Text>否认</Content_Text></NewCtrl><Section Id="预防接种史" Type="1" Reserve2=""><NewCtrl Id="数据元91" Type="3" Reserve2=""><Content_Text>预防接种史：</Content_Text></NewCtrl><NewCtrl Id="预防接种史情况" Type="6" Reserve2=""><Content_Text>未接种疫苗</Content_Text></NewCtrl><Content_Text>预防接种史：未接种疫苗</Content_Text></Section><NewCtrl Id="疫苗" Type="7" Reserve2=""><Content_Text>未接种</Content_Text></NewCtrl><Section Id="手术外伤史" Type="1" Reserve2=""><NewCtrl Id="数据元103" Type="3" Reserve2=""><Content_Text>手术外伤史：</Content_Text></NewCtrl><NewCtrl Id="手术外伤史02EED1DF1785482cA68F86345DDB6583" Type="6" Reserve2=""><Content_Text>否认手术外伤史</Content_Text></NewCtrl><Content_Text>手术外伤史：否认手术外伤史</Content_Text></Section><Section Id="输血史" Type="1" Reserve2=""><NewCtrl Id="数据元106" Type="3" Reserve2=""><Content_Text>输血史：</Content_Text></NewCtrl><Content_Text>输血史：具体输血史不详</Content_Text></Section><Section Id="过敏史" Type="1" Reserve2=""><NewCtrl Id="过敏史情况" Type="6" Reserve2=""><Content_Text>否认药物及食物过敏史</Content_Text></NewCtrl><Content_Text>药物（食物）过敏史：否认药物及食物过敏史</Content_Text></Section><Content_Text>传染病史：否认麻疹，水痘，猩红热，流行性腮腺炎，肝炎，结核等病史；否认结核接触史；

预防接种史：未接种疫苗；肺炎链球菌疫苗未接种；

手术外伤史：否认手术外伤史；

输血史：具体输血史不详；

药物（食物）过敏史：否认药物及食物过敏史 </Content_Text></Section><NewCtrl Id="

湿疹" Type="7" Reserve2=""><Content_Text>无</Content_Text></NewCtrl><NewCtrl Id="牛奶蛋白过敏" Type="7" Reserve2=""><Content_Text>无</Content_Text></NewCtrl><NewCtrl Id="过敏性鼻炎" Type="6" Reserve2=""><Content_Text>无</Content_Text></NewCtrl><NewCtrl Id="系统回顾" Type="6" Reserve2=""><Content_Text>否认</Content_Text></NewCtrl><NewCtrl Id="反复呼吸道感染" Type="6" Reserve2=""><Content_Text>否认</Content_Text></NewCtrl><NewCtrl Id="上呼吸道感染" Type="3" Reserve2=""><Content_Text>0</Content_Text></NewCtrl><NewCtrl Id="下呼吸道感染" Type="3" Reserve2=""><Content_Text>4</Content_Text></NewCtrl><NewCtrl Id="住院史" Type="6" Reserve2=""><Content_Text>有</Content_Text></NewCtrl><NewCtrl Id="住院" Type="3" Reserve2=""><Content_Text>7</Content_Text></NewCtrl><NewCtrl Id="吸氧" Type="3" Reserve2=""><Content_Text>7</Content_Text></NewCtrl><NewCtrl Id="ICU" Type="3" Reserve2=""><Content_Text>4</Content_Text></NewCtrl><NewCtrl Id="气管插管" Type="3" Reserve2=""><Content_Text>3</Content_Text></NewCtrl><NewCtrl Id="基础疾病" Type="6" Reserve2=""><Content_Text>有</Content_Text></NewCtrl><NewCtrl Id="呼吸系统疾病" Type="8" Reserve2=""><Content_Text>严重呼吸系统基础疾病，重症肺炎</Content_Text></NewCtrl><NewCtrl Id="基础疾病1" Type="7" Reserve2=""><Content_Text>有</Content_Text></NewCtrl><NewCtrl Id="其他既往疾病" Type="8" Reserve2=""><Content_Text>严重基础疾病，其他：</Content_Text></NewCtrl><NewCtrl Id="既往喘息史" Type="6" Reserve2=""><Content_Text>否认</Content_Text></NewCtrl><Section Id="个人史" Type="1" Reserve2=""><Section Id="Section_121231171514897" Type="1"

Reserve2=""><NewCtrl Id="数据元 125" Type="3" Reserve2=""><Content_Text>出生和喂养：患儿系 G</Content_Text></NewCtrl><NewCtrl Id="患儿系 G" Type="3" Reserve2=""><Content_Text>6</Content_Text></NewCtrl><NewCtrl Id="数据元 126" Type="3" Reserve2=""><Content_Text>P</Content_Text></NewCtrl><NewCtrl Id="患儿系 P" Type="3" Reserve2=""><Content_Text>3</Content_Text></NewCtrl><NewCtrl Id="数据元 127" Type="3" Reserve2=""><Content_Text>，产时</Content_Text></NewCtrl><NewCtrl Id="有无窒息" Type="7" Reserve2=""><Content_Text>有</Content_Text></NewCtrl><NewCtrl Id="数据元 128" Type="3" Reserve2=""><Content_Text>窒息，出生体重</Content_Text></NewCtrl><NewCtrl Id="出生体重" Type="3" Reserve2=""><Content_Text>1560</Content_Text></NewCtrl><NewCtrl Id="数据元 129" Type="3" Reserve2=""><Content_Text>。生后予以</Content_Text></NewCtrl><NewCtrl Id="喂养方式" Type="6" Reserve2=""><Content_Text>混合</Content_Text></NewCtrl><NewCtrl Id="数据元 130" Type="3" Reserve2=""><Content_Text>喂养，</Content_Text></NewCtrl><NewCtrl Id="添加辅食情况" Type="6" Reserve2=""><Content_Text>目前尚未添加辅食</Content_Text></NewCtrl><NewCtrl Id="数据元 132" Type="3" Reserve2=""><Content_Text>，生长发育史：</Content_Text></NewCtrl><NewCtrl Id="生长发育" Type="10" Reserve2=""><Content_Text>运动语言发育落后于正常同龄儿</Content_Text></NewCtrl><Content_Text>出生和喂养：患儿系****，****周****，产时****窒息，出生体重****。生后予以混合喂养，目前尚未添加辅食，生长发育史：运动语言发育落后于正常同龄儿。</Content_Text></Section><Content_Text>个人史：****</Content_Text></Section><Section Id="Section_Emmenia1" Type="1"

Reserve2=""><NewCtrl Id="数据元 133" Type="3" Reserve2=""><Content_Text>月经史：
</Content_Text></NewCtrl><NewCtrl Id="Section_Emmenia" Type="6"
Reserve2=""><Content_Text>无</Content_Text></NewCtrl><Content_Text>月经史：无
</Content_Text></Section><Section Id="Section_130105135824427" Type="1"
Reserve2=""><Content_Text>婚育史：无</Content_Text></Section><Section Id="家族史"
Type="1" Reserve2=""><NewCtrl Id="数据元 134" Type="3" Reserve2=""><Content_Text>
家族史：</Content_Text></NewCtrl><NewCtrl Id="数据元 135" Type="3"
Reserve2=""><Content_Text>父亲</Content_Text></NewCtrl><NewCtrl Id="父亲年龄"
Type="3" Reserve2=""><Content_Text>48</Content_Text></NewCtrl><NewCtrl Id="数据
元 137" Type="3" Reserve2=""><Content_Text>岁，职业：
</Content_Text></NewCtrl><NewCtrl Id="父亲职业" Type="3"
Reserve2=""><Content_Text>务农</Content_Text></NewCtrl><NewCtrl Id="数据元 138"
Type="3" Reserve2=""><Content_Text>，健康情况：</Content_Text></NewCtrl><NewCtrl
Id="父亲健康情况" Type="10" Reserve2=""><Content_Text>体健
</Content_Text></NewCtrl><NewCtrl Id="数据元 141" Type="3"
Reserve2=""><Content_Text>母亲</Content_Text></NewCtrl><NewCtrl Id="母亲年龄"
Type="3" Reserve2=""><Content_Text>43</Content_Text></NewCtrl><NewCtrl Id="数据
元 140" Type="3" Reserve2=""><Content_Text>岁，职业：
</Content_Text></NewCtrl><NewCtrl Id="母亲职业" Type="3"
Reserve2=""><Content_Text>务农</Content_Text></NewCtrl><NewCtrl Id="数据元 139"
Type="3" Reserve2=""><Content_Text>，健康情况：</Content_Text></NewCtrl><NewCtrl
Id="母亲健康情况" Type="10" Reserve2=""><Content_Text>体健

</Content_Text></NewCtrl><NewCtrl Id="数据元142" Type="3" Reserve2=""><Content_Text>父母近亲婚配史：</Content_Text></NewCtrl><NewCtrl Id="婚配情况" Type="6" Reserve2=""><Content_Text>否认父母近亲婚配</Content_Text></NewCtrl><NewCtrl Id="数据元143" Type="3" Reserve2=""><Content_Text>家庭其他成员健康情况：</Content_Text></NewCtrl><Content_Text>家族史：

父亲****岁，职业：****，健康情况：****；

母亲****岁，职业：****，健康情况：****；

大姐****岁，职业：务工，健康情况：****；

二姐****岁，职业：上学，健康情况：****；

父母近亲婚配史：否认父母近亲婚配；

家庭其他成员健康情况：****；

</Content_Text></Section><NewCtrl Id="吸烟暴露" Type="6" Reserve2=""><Content_Text>有</Content_Text></NewCtrl><Section Id="体格检查" Type="1" Reserve2=""><Section Id="section12311" Type="1" Reserve2=""><NewCtrl Id="数据元144" Type="3" Reserve2=""><Content_Text>体格检查：</Content_Text></NewCtrl><Section Id="生命体征" Type="1" Reserve2=""><NewCtrl Id="数据元145" Type="3" Reserve2=""><Content_Text>体温</Content_Text></NewCtrl><NewCtrl Id="体温测量方式" Type="7" Reserve2=""><Content_Text>耳温</Content_Text></NewCtrl><NewCtrl Id="数据元146" Type="3" Reserve2=""><Content_Text>℃</Content_Text></NewCtrl><NewCtrl Id="数据元147" Type="3" Reserve2=""><Content_Text>；脉搏</Content_Text></NewCtrl><NewCtrl Id="脉

搏次数" Type="5" Reserve2=""><Content_Text>129</Content_Text></NewCtrl><NewCtrl Id="数据元 149" Type="3" Reserve2=""><Content_Text>次/分</Content_Text></NewCtrl><NewCtrl Id="数据元 148" Type="3" Reserve2=""><Content_Text>；呼吸</Content_Text></NewCtrl><NewCtrl Id="呼吸次数值" Type="5" Reserve2=""><Content_Text>30</Content_Text></NewCtrl><NewCtrl Id="数据元 150" Type="3" Reserve2=""><Content_Text>次/分</Content_Text></NewCtrl><NewCtrl Id="数据元 151" Type="3" Reserve2=""><Content_Text>；血压</Content_Text></NewCtrl><NewCtrl Id="血压前值" Type="5" Reserve2=""><Content_Text>74</Content_Text></NewCtrl><NewCtrl Id="血压后值" Type="5" Reserve2=""><Content_Text>34</Content_Text></NewCtrl><NewCtrl Id="数据元 152" Type="3" Reserve2=""><Content_Text>mmHg</Content_Text></NewCtrl><NewCtrl Id="数据元 153" Type="3" Reserve2=""><Content_Text>; 身长/高</Content_Text></NewCtrl><NewCtrl Id="身长高值" Type="5" Reserve2=""><Content_Text>64</Content_Text></NewCtrl><NewCtrl Id="数据元 154" Type="3" Reserve2=""><Content_Text>cm</Content_Text></NewCtrl><NewCtrl Id="数据元 155" Type="3" Reserve2=""><Content_Text>；体重</Content_Text></NewCtrl><NewCtrl Id="体重值" Type="5" Reserve2=""><Content_Text>5.5</Content_Text></NewCtrl><NewCtrl Id="数据元 156" Type="3" Reserve2=""><Content_Text>kg</Content_Text></NewCtrl><NewCtrl Id="氧合" Type="5" Reserve2=""><Content_Text>98</Content_Text></NewCtrl><Content_Text>体温耳温*****℃；脉搏****次/分；呼吸****次/分；血压****mmHg；身长/高****cm；体重

****kg；未吸氧下血氧饱和度****%；

</Content_Text></Section><Section Id="Section_121231175511874" Type="1" Reserve2=""><NewCtrl Id="数据元 164" Type="3" Reserve2=""><Content_Text>【一般情况】</Content_Text></NewCtrl><Section Id="一般情况" Type="1" Reserve2=""><NewCtrl Id="数据元 170" Type="3" Reserve2=""><Content_Text>神志</Content_Text></NewCtrl><NewCtrl Id="神志" Type="6" Reserve2=""><Content_Text>清</Content_Text></NewCtrl><NewCtrl Id="数据元 173" Type="3" Reserve2=""><Content_Text>，精神反应</Content_Text></NewCtrl><NewCtrl Id="精神反应情况" Type="6" Reserve2=""><Content_Text>良好</Content_Text></NewCtrl><NewCtrl Id="数据元 182" Type="3" Reserve2=""><Content_Text>，发育</Content_Text></NewCtrl><NewCtrl Id="发育情况" Type="6" Reserve2=""><Content_Text>正常</Content_Text></NewCtrl><NewCtrl Id="数据元 184" Type="3" Reserve2=""><Content_Text>，面色</Content_Text></NewCtrl><NewCtrl Id="面色情况" Type="6" Reserve2=""><Content_Text>正常</Content_Text></NewCtrl><NewCtrl Id="数据元 185" Type="3" Reserve2=""><Content_Text>营养：</Content_Text></NewCtrl><NewCtrl Id="营养情况" Type="7" Reserve2=""><Content_Text>中等</Content_Text></NewCtrl><Content_Text>神志清，精神反应良好，发育正常，面色正常，营养：中等。</Content_Text></Section><Content_Text>【一般情况】神志清，精神反应良好，发育正常，面色正常，营养：中等。</Content_Text></Section><Section Id="皮肤黏膜" Type="1" Reserve2=""><NewCtrl Id="数据元 186" Type="3" Reserve2=""><Content_Text>【皮肤、黏膜】</Content_Text></NewCtrl><NewCtrl Id="数据元 187" Type="3"

Reserve2=""><Content_Text>皮肤弹性</Content_Text></NewCtrl><NewCtrl Id="皮肤弹性" Type="6" Reserve2=""><Content_Text>良好</Content_Text></NewCtrl><NewCtrl Id="数据元 188" Type="3" Reserve2=""><Content_Text>，四肢末梢</Content_Text></NewCtrl><NewCtrl Id="四肢末梢" Type="6" Reserve2=""><Content_Text>暖</Content_Text></NewCtrl><NewCtrl Id="有无苍白" Type="7" Reserve2=""><Content_Text>无</Content_Text></NewCtrl><NewCtrl Id="数据元 189" Type="3" Reserve2=""><Content_Text>苍白</Content_Text></NewCtrl><NewCtrl Id="黄染程度" Type="7" Reserve2=""><Content_Text>无</Content_Text></NewCtrl><NewCtrl Id="数据元 190" Type="3" Reserve2=""><Content_Text>黄染</Content_Text></NewCtrl><NewCtrl Id="青紫程度" Type="7" Reserve2=""><Content_Text>无</Content_Text></NewCtrl><NewCtrl Id="数据元 192" Type="3" Reserve2=""><Content_Text>青紫</Content_Text></NewCtrl><NewCtrl Id="出血点" Type="6" Reserve2=""><Content_Text>未见</Content_Text></NewCtrl><NewCtrl Id="数据元 193" Type="3" Reserve2=""><Content_Text>出血点</Content_Text></NewCtrl><NewCtrl Id="皮疹" Type="6" Reserve2=""><Content_Text>无</Content_Text></NewCtrl><NewCtrl Id="数据元 194" Type="3" Reserve2=""><Content_Text>皮疹</Content_Text></NewCtrl><NewCtrl Id="毛细血管再充盈时间" Type="3" Reserve2=""><Content_Text>毛细血管再充盈时间</Content_Text></NewCtrl><Content_Text>【皮肤、黏膜】皮肤弹性良好，四肢末梢暖，面色无苍白、无黄染、无青紫，未见出血点、无皮疹。毛细血管再充盈时间</Content_Text></Section><NewCtrl Id="CRT" Type="7" Reserve2=""><Content_Text>≤2s</Content_Text></NewCtrl><Section Id="浅表淋巴结"

Type="1" Reserve2=""><NewCtrl Id="数据元 195" Type="3" Reserve2=""><Content_Text>【浅表淋巴结】</Content_Text></NewCtrl><Section Id="section130403183805" Type="1" Reserve2=""><NewCtrl Id="浅表淋巴结情况" Type="6" Reserve2=""><Content_Text>浅表淋巴结未及</Content_Text></NewCtrl><Content_Text>浅表淋巴结未及。</Content_Text></Section><Content_Text>【浅表淋巴结】浅表淋巴结未及。</Content_Text></Section><Section Id="头部及其器官" Type="1" Reserve2=""><NewCtrl Id="数据元 196" Type="3" Reserve2=""><Content_Text>【头部及其器官】</Content_Text></NewCtrl><Section Id="颅" Type="1" Reserve2=""><NewCtrl Id="数据元 197" Type="3" Reserve2=""><Content_Text>颅：外形</Content_Text></NewCtrl><NewCtrl Id="外形" Type="6" Reserve2=""><Content_Text>正常</Content_Text></NewCtrl><NewCtrl Id="数据元 198" Type="3" Reserve2=""><Content_Text>头发</Content_Text></NewCtrl><NewCtrl Id="头发" Type="6" Reserve2=""><Content_Text>分布均匀</Content_Text></NewCtrl><NewCtrl Id="特殊面容" Type="6" Reserve2=""><Content_Text>无特殊面容</Content_Text></NewCtrl><NewCtrl Id="囟门情况" Type="7" Reserve2=""><Content_Text>已闭</Content_Text></NewCtrl><Content_Text>颅：外形正常，头发分布均匀，无特殊面容。囟门已闭</Content_Text></Section><Section Id="眼" Type="1" Reserve2=""><NewCtrl Id="数据元 199" Type="3" Reserve2=""><Content_Text>眼：眼睑</Content_Text></NewCtrl><NewCtrl Id="眼睑浮肿下垂程度" Type="6" Reserve2=""><Content_Text>无明显</Content_Text></NewCtrl><NewCtrl Id="眼睑浮肿下垂" Type="6" Reserve2=""><Content_Text>浮肿</Content_Text></NewCtrl><NewCtrl Id="数据元 200" Type="3" Reserve2=""><Content_Text>结膜

</Content_Text></NewCtrl><NewCtrl Id="结膜充血情况" Type="6" Reserve2=""><Content_Text>无充血</Content_Text></NewCtrl><NewCtrl Id="数据元 203" Type="3" Reserve2=""><Content_Text>巩膜</Content_Text></NewCtrl><NewCtrl Id="巩膜黄染" Type="6" Reserve2=""><Content_Text>无</Content_Text></NewCtrl><NewCtrl Id="数据元 204" Type="3" Reserve2=""><Content_Text>黄染</Content_Text></NewCtrl><NewCtrl Id="数据元 205" Type="3" Reserve2=""><Content_Text>眼球</Content_Text></NewCtrl><NewCtrl Id="眼球情况" Type="6" Reserve2=""><Content_Text>活动自如</Content_Text></NewCtrl><NewCtrl Id="数据元 206" Type="3" Reserve2=""><Content_Text>双瞳孔</Content_Text></NewCtrl><NewCtrl Id="瞳孔" Type="8" Reserve2=""><Content_Text>等大等圆</Content_Text></NewCtrl><NewCtrl Id="数据元 207" Type="3" Reserve2=""><Content_Text>对光反射</Content_Text></NewCtrl><NewCtrl Id="对光反射" Type="7" Reserve2=""><Content_Text>存在</Content_Text></NewCtrl><Content_Text>眼：眼睑无明显浮肿，结膜无充血，巩膜无黄染，眼球活动自如，双瞳孔等大等圆，对光反射存在。</Content_Text></Section><Section Id="耳" Type="1" Reserve2=""><NewCtrl Id="数据元 208" Type="3" Reserve2=""><Content_Text>耳：耳廓</Content_Text></NewCtrl><NewCtrl Id="耳廓外观" Type="6" Reserve2=""><Content_Text>外观无异常</Content_Text></NewCtrl><NewCtrl Id="数据元 209" Type="3" Reserve2=""><Content_Text>外耳道</Content_Text></NewCtrl><NewCtrl Id="外耳道分泌物情况" Type="7" Reserve2=""><Content_Text>无</Content_Text></NewCtrl><NewCtrl Id="数据元 210" Type="3" Reserve2=""><Content_Text>分泌物

</Content_Text></NewCtrl><NewCtrl Id="数据元212" Type="3" Reserve2=""><Content_Text>乳突</Content_Text></NewCtrl><NewCtrl Id="乳突压痛" Type="7" Reserve2=""><Content_Text>无明显</Content_Text></NewCtrl><NewCtrl Id="数据元213" Type="3" Reserve2=""><Content_Text>压痛</Content_Text></NewCtrl><Content_Text>耳：耳廓外观无异常，外耳道无分泌物。乳突无明显压痛。</Content_Text></Section><Section Id="鼻" Type="1" Reserve2=""><NewCtrl Id="数据元214" Type="3" Reserve2=""><Content_Text>鼻：</Content_Text></NewCtrl><NewCtrl Id="鼻道" Type="6" Reserve2=""><Content_Text>鼻道通畅</Content_Text></NewCtrl><NewCtrl Id="数据元215" Type="3" Reserve2=""><Content_Text>鼻中隔</Content_Text></NewCtrl><NewCtrl Id="鼻中隔" Type="6" Reserve2=""><Content_Text>居中</Content_Text></NewCtrl><NewCtrl Id="鼻窦压痛" Type="7" Reserve2=""><Content_Text>无</Content_Text></NewCtrl><Content_Text>鼻：鼻道通畅，鼻中隔居中,鼻窦无压痛。</Content_Text></Section><NewCtrl Id="数据元216" Type="3" Reserve2=""><Content_Text>口腔及咽部：</Content_Text></NewCtrl><Section Id="口腔及咽部" Type="1" Reserve2=""><NewCtrl Id="数据元217" Type="3" Reserve2=""><Content_Text>口唇</Content_Text></NewCtrl><NewCtrl Id="口唇" Type="10" Reserve2=""><Content_Text>无干燥</Content_Text></NewCtrl><NewCtrl Id="数据元218" Type="3" Reserve2=""><Content_Text>咽</Content_Text></NewCtrl><NewCtrl Id="咽" Type="7" Reserve2=""><Content_Text>不红</Content_Text></NewCtrl><NewCtrl Id="数据元219" Type="3" Reserve2=""><Content_Text>扁桃体</Content_Text></NewCtrl><NewCtrl Id="数据元

220" Type="3" Reserve2=""><Content_Text>口腔黏膜</Content_Text></NewCtrl><NewCtrl Id="口腔黏膜" Type="6" Reserve2=""><Content_Text>完整</Content_Text></NewCtrl><Content_Text>口唇无干燥，咽不红，扁桃体稍红，无渗出，口腔黏膜完整。</Content_Text></Section><Content_Text>

【头部及其器官】

颅：外形正常，头发分布均匀，无特殊面容。囟门已闭；

眼：眼睑无明显浮肿，结膜无充血，巩膜无黄染，眼球活动自如，双瞳孔等大等圆，对光反射存在。

耳：耳廓外观无异常，外耳道无分泌物。乳突无明显压痛。

鼻：鼻道通畅，鼻中隔居中,鼻窦无压痛。

口腔及咽部：口唇无干燥，咽不红，扁桃体稍红，无渗出，口腔黏膜完整。</Content_Text></Section><Section Id="颈部" Type="1" Reserve2=""><NewCtrl Id="数据元 221" Type="3" Reserve2=""><Content_Text>【颈部】</Content_Text></NewCtrl><NewCtrl Id="颈亢情况" Type="7" Reserve2=""><Content_Text>无</Content_Text></NewCtrl><NewCtrl Id="数据元 222" Type="3" Reserve2=""><Content_Text>颈亢，颈静脉</Content_Text></NewCtrl><NewCtrl Id="颈静脉怒张情况" Type="6" Reserve2=""><Content_Text>无</Content_Text></NewCtrl><NewCtrl Id="数据元 223" Type="3" Reserve2=""><Content_Text>怒张，气管</Content_Text></NewCtrl><NewCtrl Id="气管" Type="6" Reserve2=""><Content_Text>居中</Content_Text></NewCtrl><Content_Text>【颈部】无颈亢，颈静脉无怒张，气管居中。</Content_Text></Section><Section Id="胸部" Type="1" Reserve2=""><NewCtrl Id="胸部对称" Type="7" Reserve2=""><Content_Text>

对称</Content_Text></NewCtrl><NewCtrl Id="胸部有无畸形" Type="7" Reserve2=""><Content_Text>无</Content_Text></NewCtrl><NewCtrl Id="数据元2251" Type="3" Reserve2=""><Content_Text>畸形，胸壁</Content_Text></NewCtrl><NewCtrl Id="胸部有无" Type="7" Reserve2=""><Content_Text>无</Content_Text></NewCtrl><NewCtrl Id="胸壁情况" Type="6" Reserve2=""><Content_Text>皮下气肿</Content_Text></NewCtrl><NewCtrl Id="胸廓畸形" Type="7" Reserve2=""><Content_Text>无</Content_Text></NewCtrl><NewCtrl Id="胸廓" Type="8" Reserve2=""><Content_Text>胸廓畸形</Content_Text></NewCtrl><NewCtrl Id="数据元226" Type="3" Reserve2=""><Content_Text>心</Content_Text></NewCtrl><Section Id="心望诊" Type="1" Reserve2=""><NewCtrl Id="数据元227" Type="3" Reserve2=""><Content_Text>望诊：心前区</Content_Text></NewCtrl><NewCtrl Id="心前区情况" Type="6" Reserve2=""><Content_Text>无隆起</Content_Text></NewCtrl><Content_Text>望诊：心前区无隆起。</Content_Text></Section><Section Id="心触诊" Type="1" Reserve2=""><NewCtrl Id="数据元228" Type="3" Reserve2=""><Content_Text>触诊：心前区</Content_Text></NewCtrl><NewCtrl Id="心尖部有无震颤及情况" Type="6" Reserve2=""><Content_Text>无震颤</Content_Text></NewCtrl><Content_Text>触诊：心前区无震颤。</Content_Text></Section><Section Id="Section_KouZheng" Type="1" Reserve2=""><NewCtrl Id="数据元4" Type="3" Reserve2=""><Content_Text>叩诊:</Content_Text></NewCtrl><NewCtrl Id="数据元232" Type="3" Reserve2=""><Content_Text>右(cm)</Content_Text></NewCtrl><NewCtrl Id="数据元5" Type="3" Reserve2=""><Content_Text>肋间</Content_Text></NewCtrl><NewCtrl Id="数

据元234" Type="3" Reserve2=""><Content_Text>左(cm)</Content_Text></NewCtrl><NewCtrl Id="数据元230" Type="3" Reserve2=""><Content_Text>左锁骨中线距正中线</Content_Text></NewCtrl><NewCtrl Id="左锁骨中线距正中线" Type="5" Reserve2=""><Content_Text>3</Content_Text></NewCtrl><NewCtrl Id="数据元231" Type="3" Reserve2=""><Content_Text>cm</Content_Text></NewCtrl><NewCtrl Id="数据元7" Type="3" Reserve2=""><Content_Text>心界</Content_Text></NewCtrl><NewCtrl Id="心界有无扩大" Type="6" Reserve2=""><Content_Text>无</Content_Text></NewCtrl><NewCtrl Id="数据元236" Type="3" Reserve2=""><Content_Text>扩大。</Content_Text></NewCtrl><Content_Text>

5 岁以下不用叩心界

叩诊:

右(cm)

肋间

左(cm)

II

III

IV

V

左锁骨中线距正中线 3 cm

心界无扩大。

</Content_Text></Section><Content_Text>【胸部】胸部对称，无畸形，胸壁无皮下气

肿。无胸廓畸形。

心　望诊：心前区无隆起。

触诊：心前区无震颤。

5 岁以下不用叩心界

叩诊:

右(cm)

肋间

左(cm)

II

III

IV

V

左锁骨中线距正中线 3 cm

心界无扩大。

</Content_Text></Section><Section Id="心听诊" Type="1" Reserve2=""><NewCtrl Id="数据元 2371" Type="3" Reserve2=""><Content_Text>听诊：心率</Content_Text></NewCtrl><NewCtrl Id="心率 1" Type="5" Reserve2=""><Content_Text>129</Content_Text></NewCtrl><NewCtrl Id="数据元 2381" Type="3" Reserve2=""><Content_Text>次/分</Content_Text></NewCtrl><NewCtrl Id="心律情况 1" Type="6" Reserve2=""><Content_Text>心律齐</Content_Text></NewCtrl><NewCtrl Id="心音情况 1" Type="6" Reserve2=""><Content_Text>心音有力</Content_Text></NewCtrl><NewCtrl Id="杂音情

况 1" Type="6" Reserve2=""><Content_Text>未及杂音</Content_Text></NewCtrl><Content_Text>听诊：心率 129 次/分，心律齐，心音有力，未及杂音。</Content_Text></Section><Section Id="肺" Type="1" Reserve2=""><Section Id="肺望诊" Type="1" Reserve2=""><NewCtrl Id="数据元 239" Type="3" Reserve2=""><Content_Text>肺 望诊：</Content_Text></NewCtrl><NewCtrl Id="肺望诊情况" Type="6" Reserve2=""><Content_Text>双侧呼吸运动对称</Content_Text></NewCtrl><NewCtrl Id="有无呼吸困难" Type="6" Reserve2=""><Content_Text>有吸气时</Content_Text></NewCtrl><NewCtrl Id="呼吸困难表现" Type="10" Reserve2=""><Content_Text>锁骨上凹</Content_Text></NewCtrl><NewCtrl Id="呼吸频率 1" Type="5" Reserve2=""><Content_Text>30</Content_Text></NewCtrl><NewCtrl Id="有无呼吸增快" Type="6" Reserve2=""><Content_Text>无</Content_Text></NewCtrl><Content_Text>肺

望诊：双侧呼吸运动对称。 有吸气时锁骨上凹。

呼吸频率：30 次/min，无</Content_Text></Section><Section Id="肺触诊" Type="1" Reserve2=""><NewCtrl Id="数据元 240" Type="3" Reserve2=""><Content_Text>触诊：</Content_Text></NewCtrl><NewCtrl Id="哭颤语颤" Type="7" Reserve2=""><Content_Text>语颤</Content_Text></NewCtrl><NewCtrl Id="肺触诊是否对称" Type="6" Reserve2=""><Content_Text>对称</Content_Text></NewCtrl><NewCtrl Id="有无杂音" Type="6" Reserve2=""><Content_Text>无</Content_Text></NewCtrl><NewCtrl Id="肺部触诊" Type="10" Reserve2=""><Content_Text>胸膜摩擦感，握雪感</Content_Text></NewCtrl><Content_Text>触诊：语颤对称，无胸膜摩擦感，握雪感。

</Content_Text></Section><Section Id="肺叩诊" Type="1" Reserve2=""><NewCtrl Id="数据元 241" Type="3" Reserve2=""><Content_Text>叩诊：</Content_Text></NewCtrl><NewCtrl Id="肺叩诊是否对称" Type="6" Reserve2=""><Content_Text>双侧对称</Content_Text></NewCtrl><NewCtrl Id="性质" Type="6" Reserve2=""><Content_Text>清音</Content_Text></NewCtrl><Content_Text>叩诊：双侧对称，呈清音。</Content_Text></Section><Section Id="肺听诊" Type="1" Reserve2=""><NewCtrl Id="数据元 242" Type="3" Reserve2=""><Content_Text>听诊：双侧呼吸音</Content_Text></NewCtrl><NewCtrl Id="双侧呼吸音情况" Type="6" Reserve2=""><Content_Text>粗</Content_Text></NewCtrl><NewCtrl Id="肺的部位" Type="10" Reserve2=""><Content_Text>双肺</Content_Text></NewCtrl><NewCtrl Id="呼吸音" Type="7" Reserve2=""><Content_Text>对称</Content_Text></NewCtrl><NewCtrl Id="肺的部位 1" Type="10" Reserve2=""><Content_Text>双肺</Content_Text></NewCtrl><NewCtrl Id="是否有湿罗音" Type="7" Reserve2=""><Content_Text>可及</Content_Text></NewCtrl><NewCtrl Id="湿罗音" Type="7" Reserve2=""><Content_Text>粗湿啰音</Content_Text></NewCtrl><NewCtrl Id="肺的部位 11" Type="10" Reserve2=""><Content_Text>双肺</Content_Text></NewCtrl><NewCtrl Id="是否有哮鸣音 1" Type="7" Reserve2=""><Content_Text>未及</Content_Text></NewCtrl><NewCtrl Id="哮鸣音 1" Type="7" Reserve2=""><Content_Text>干啰音</Content_Text></NewCtrl><Content_Text>听诊：双侧呼吸音粗，双肺呼吸音对称，双肺可及粗湿啰音，双肺未及干啰音。</Content_Text></Section><Content_Text>肺　　望诊：双侧呼吸运动对称。有吸气时锁骨上凹。

呼吸频率：30 次/min，无呼吸增快（平静时观察 1 min：小于 2 月龄≥60 次 / min；2 月龄 ~ 1 岁≥50 次 / min ； 1 ~ 5 岁≥40 次 / min；5 岁以上≥30 次 / min）。

触诊：语颤对称，无胸膜摩擦感，握雪感。

叩诊：双侧对称，呈清音。

听诊：双侧呼吸音粗，双肺呼吸音对称，双肺可及粗湿啰音，双肺未及干啰音。</Content_Text></Section><Section Id="血管" Type="1" Reserve2=""><NewCtrl Id="数据元244" Type="3" Reserve2=""><Content_Text>【血管】</Content_Text></NewCtrl><NewCtrl Id="周围血管征" Type="6" Reserve2=""><Content_Text>无毛细血管搏动征和枪击音</Content_Text></NewCtrl><Content_Text>【血管】无毛细血管搏动征和枪击音。</Content_Text></Section><NewCtrl Id="数据元245" Type="3" Reserve2=""><Content_Text>【腹部】</Content_Text></NewCtrl><Section Id="腹部" Type="1" Reserve2=""><Section Id="腹部望诊 1" Type="1" Reserve2=""><NewCtrl Id="数据元2461" Type="3" Reserve2=""><Content_Text>望诊：</Content_Text></NewCtrl><NewCtrl Id="腹部望诊外形" Type="6" Reserve2=""><Content_Text>平坦</Content_Text></NewCtrl><NewCtrl Id="肠型 1" Type="6" Reserve2=""><Content_Text>未见</Content_Text></NewCtrl><NewCtrl Id="数据元2471" Type="3" Reserve2=""><Content_Text>肠型。</Content_Text></NewCtrl><NewCtrl Id="腹部静脉 1" Type="6" Reserve2=""><Content_Text>腹壁静脉未见曲张</Content_Text></NewCtrl><NewCtrl Id="脐疝" Type="7" Reserve2=""><Content_Text>无</Content_Text></NewCtrl><Content_Text>望诊：平坦，未见肠型。腹壁静脉未见曲张。

无</Content_Text></Section><Section Id="腹部触诊 1" Type="1" Reserve2=""><NewCtrl Id="数据元 2481" Type="3" Reserve2=""><Content_Text>触诊：</Content_Text></NewCtrl><NewCtrl Id="腹部触诊情况 1" Type="6" Reserve2=""><Content_Text>全腹软</Content_Text></NewCtrl><NewCtrl Id="有无包块 1" Type="7" Reserve2=""><Content_Text>未及</Content_Text></NewCtrl><NewCtrl Id="数据元 2491" Type="3" Reserve2=""><Content_Text>包块。</Content_Text></NewCtrl><NewCtrl Id="压痛与跳痛情况 1" Type="6" Reserve2=""><Content_Text>无压痛，无反跳痛</Content_Text></NewCtrl><NewCtrl Id="数据元 2501" Type="3" Reserve2=""><Content_Text>肝脏：肋下</Content_Text></NewCtrl><NewCtrl Id="肝脏肋下大小 1" Type="6" Reserve2=""><Content_Text>未触及</Content_Text></NewCtrl><NewCtrl Id="数据元 2511" Type="3" Reserve2=""><Content_Text>剑突下</Content_Text></NewCtrl><NewCtrl Id="肝脏剑突下大小 1" Type="6" Reserve2=""><Content_Text>未触及</Content_Text></NewCtrl><NewCtrl Id="肝质地" Type="7" Reserve2=""><Content_Text>韧</Content_Text></NewCtrl><NewCtrl Id="数据元 2521" Type="3" Reserve2=""><Content_Text>脾脏：肋下</Content_Text></NewCtrl><NewCtrl Id="脾脏肋下大小 1" Type="6" Reserve2=""><Content_Text>未触及</Content_Text></NewCtrl><NewCtrl Id="肝质地 1" Type="7" Reserve2=""><Content_Text>韧</Content_Text></NewCtrl><Content_Text>触诊：全腹软，未及包块。无压痛，无反跳痛。

肝脏：肋下未触及，剑突下未触及，质地韧。

脾脏：肋下未触及，质地韧。</Content_Text></Section><Section Id="腹部

叩诊 1" Type="1" Reserve2=""><NewCtrl Id="数据元 2531" Type="3" Reserve2=""><Content_Text>叩诊：</Content_Text></NewCtrl><NewCtrl Id="腹部叩诊性质" Type="6" Reserve2=""><Content_Text>鼓音</Content_Text></NewCtrl><NewCtrl Id="有无移动性浊音 1" Type="7" Reserve2=""><Content_Text>无</Content_Text></NewCtrl><NewCtrl Id="数据元 2541" Type="3" Reserve2=""><Content_Text>移动性浊音。</Content_Text></NewCtrl><Content_Text>叩诊：鼓音，无移动性浊音。</Content_Text></Section><Section Id="腹部听诊 1" Type="1" Reserve2=""><NewCtrl Id="数据元 2551" Type="3" Reserve2=""><Content_Text>听诊：肠鸣音</Content_Text></NewCtrl><NewCtrl Id="肠鸣音" Type="7" Reserve2=""><Content_Text>正常</Content_Text></NewCtrl><Content_Text>听诊：肠鸣音正常。</Content_Text></Section><Content_Text>　　望诊：平坦，未见肠型。腹壁静脉未见曲张。无脐疝。

触诊：全腹软，未及包块。无压痛，无反跳痛。

肝脏：肋下未触及，剑突下未触及，质地韧。

脾脏：肋下未触及，质地韧。

叩诊：鼓音，无移动性浊音。

听诊：肠鸣音正常。　　</Content_Text></Section><Section Id="脊柱四肢" Type="1" Reserve2=""><NewCtrl Id="数据元 257" Type="3" Reserve2=""><Content_Text>【脊柱四肢】</Content_Text></NewCtrl><NewCtrl Id="有无畸形" Type="6" Reserve2=""><Content_Text>无</Content_Text></NewCtrl><NewCtrl Id="数据元 258" Type="3" Reserve2=""><Content_Text>畸形，活动</Content_Text></NewCtrl><NewCtrl Id="活动情况" Type="6" Reserve2=""><Content_Text>自如

</Content_Text></NewCtrl><NewCtrl Id="有无关节红肿" Type="6" Reserve2=""><Content_Text>无关节红肿</Content_Text></NewCtrl><Content_Text>【脊柱四肢】无畸形，活动自如，无关节红肿。</Content_Text></Section><Section Id="直肠肛门" Type="1" Reserve2=""><NewCtrl Id="数据元 259" Type="3" Reserve2=""><Content_Text>【直肠肛门】</Content_Text></NewCtrl><NewCtrl Id="肛门情况" Type="6" Reserve2=""><Content_Text>肛门存在</Content_Text></NewCtrl><NewCtrl Id="肛裂情况" Type="6" Reserve2=""><Content_Text>未见肛裂</Content_Text></NewCtrl><Content_Text>【直肠肛门】肛门存在，未见肛裂。</Content_Text></Section><Section Id="外生殖器" Type="1" Reserve2=""><NewCtrl Id="数据元 260" Type="3" Reserve2=""><Content_Text>【外生殖器】</Content_Text></NewCtrl><NewCtrl Id="外生殖器情况" Type="6" Reserve2=""><Content_Text>正常外生殖器</Content_Text></NewCtrl><Content_Text>【外生殖器】正常外生殖器。</Content_Text></Section><NewCtrl Id="斜疝" Type="7" Reserve2=""><Content_Text>无</Content_Text></NewCtrl><NewCtrl Id="数据元 2601" Type="3" Reserve2=""><Content_Text>【神经系统】</Content_Text></NewCtrl><Section Id="体格检查神经系统" Type="1" Reserve2=""><NewCtrl Id="数据元 261" Type="3" Reserve2=""><Content_Text>颈</Content_Text></NewCtrl><NewCtrl Id="有无颈亢" Type="7" Reserve2=""><Content_Text>软</Content_Text></NewCtrl><NewCtrl Id="数据元 262" Type="3" Reserve2=""><Content_Text>，布氏征</Content_Text></NewCtrl><NewCtrl Id="布氏征情况" Type="7" Reserve2=""><Content_Text>阴性</Content_Text></NewCtrl><NewCtrl Id="数据元 263" Type="3" Reserve2=""><Content_Text>克氏征</Content_Text></NewCtrl><NewCtrl Id="

克氏症情况" Type="7" Reserve2=""><Content_Text>阴性</Content_Text></NewCtrl><NewCtrl Id="数据元 264" Type="3" Reserve2=""><Content_Text>膝反射</Content_Text></NewCtrl><NewCtrl Id="膝反射情况" Type="6" Reserve2=""><Content_Text>正常</Content_Text></NewCtrl><NewCtrl Id="数据元 265" Type="3" Reserve2=""><Content_Text>踺反射</Content_Text></NewCtrl><NewCtrl Id="踺反射情况" Type="6" Reserve2=""><Content_Text>正常</Content_Text></NewCtrl><NewCtrl Id="数据元 266" Type="3" Reserve2=""><Content_Text>巴氏征</Content_Text></NewCtrl><NewCtrl Id="巴氏征情况" Type="6" Reserve2=""><Content_Text>阴性</Content_Text></NewCtrl><NewCtrl Id="肌张力" Type="7" Reserve2=""><Content_Text>正常</Content_Text></NewCtrl><Content_Text>颈软，布氏征阴性，克氏征阴性，膝反射正常，踺反射正常。巴氏征阴性，四肢肌张力正常，四肢肌力</Content_Text></Section><Content_Text>体格检查：****</Content_Text></Section><NewCtrl Id="数据元 110" Type="12" Reserve2=""><Content_Text /></NewCtrl><NewCtrl Id="数据元 229" Type="12" Reserve2=""><Content_Text /></NewCtrl><NewCtrl Id="数据元 310" Type="12" Reserve2=""><Content_Text /></NewCtrl><NewCtrl Id="数据元 47" Type="12" Reserve2=""><Content_Text /></NewCtrl><NewCtrl Id="数据元 52" Type="12" Reserve2=""><Content_Text /></NewCtrl><NewCtrl Id="数据元 65" Type="12" Reserve2=""><Content_Text /></NewCtrl><NewCtrl Id="数据元 611" Type="12" Reserve2=""><Content_Text /></NewCtrl><NewCtrl Id="数据元 621" Type="12" Reserve2=""><Content_Text /></NewCtrl><NewCtrl Id="数据元 631" Type="12"

Reserve2=""><Content_Text /></NewCtrl><NewCtrl Id="数据元641" Type="12"
Reserve2=""><Content_Text /></NewCtrl><NewCtrl Id="数据元651" Type="12"
Reserve2=""><Content_Text /></NewCtrl><NewCtrl Id="数据元661" Type="12"
Reserve2=""><Content_Text /></NewCtrl><NewCtrl Id="数据元671" Type="12"
Reserve2=""><Content_Text /></NewCtrl><NewCtrl Id="数据元681" Type="12"
Reserve2=""><Content_Text /></NewCtrl><NewCtrl Id="数据元69" Type="12"
Reserve2=""><Content_Text /></NewCtrl><NewCtrl Id="数据元610" Type="12"
Reserve2=""><Content_Text /></NewCtrl><NewCtrl Id="数据元6111" Type="12"
Reserve2=""><Content_Text /></NewCtrl><NewCtrl Id="数据元612" Type="12"
Reserve2=""><Content_Text /></NewCtrl><NewCtrl Id="数据元613" Type="12"
Reserve2=""><Content_Text /></NewCtrl><NewCtrl Id="数据元614" Type="12"
Reserve2=""><Content_Text /></NewCtrl><NewCtrl Id="数据元615" Type="12"
Reserve2=""><Content_Text /></NewCtrl><NewCtrl Id="数据元616" Type="12"
Reserve2=""><Content_Text /></NewCtrl><NewCtrl Id="数据元617" Type="12"
Reserve2=""><Content_Text /></NewCtrl><NewCtrl Id="数据元618" Type="12"
Reserve2=""><Content_Text /></NewCtrl><NewCtrl Id="数据元619" Type="12"
Reserve2=""><Content_Text /></NewCtrl><NewCtrl Id="数据元620" Type="12"
Reserve2=""><Content_Text /></NewCtrl><NewCtrl Id="数据元6211" Type="12"
Reserve2=""><Content_Text /></NewCtrl><NewCtrl Id="数据元622" Type="12"
Reserve2=""><Content_Text /></NewCtrl><NewCtrl Id="数据元623" Type="12"
Reserve2=""><Content_Text /></NewCtrl><NewCtrl Id="数据元624" Type="12"
Reserve2=""><Content_Text /></NewCtrl><NewCtrl Id="数据元625" Type="12"

Reserve2=""><Content_Text /></NewCtrl><NewCtrl Id="数据元 626" Type="12" Reserve2=""><Content_Text /></NewCtrl><NewCtrl Id="数据元 627" Type="12" Reserve2=""><Content_Text /></NewCtrl><NewCtrl Id="数据元 628" Type="12" Reserve2=""><Content_Text /></NewCtrl><NewCtrl Id="数据元 629" Type="12" Reserve2=""><Content_Text /></NewCtrl><NewCtrl Id="肺炎评分" Type="3" Reserve2=""><Content_Text>7</Content_Text></NewCtrl><NewCtrl Id="肺的部位 12" Type="10" Reserve2=""><Content_Text>双肺</Content_Text></NewCtrl><NewCtrl Id="是否有湿罗音 1" Type="7" Reserve2=""><Content_Text>可及</Content_Text></NewCtrl><NewCtrl Id="啰音" Type="7" Reserve2=""><Content_Text>粗湿啰音</Content_Text></NewCtrl><NewCtrl Id="数据元 268" Type="3" Reserve2=""><Content_Text>10.辅助检查：</Content_Text></NewCtrl><Section Id="辅助检查" Type="1" Reserve2=""><Section Id="专科检查 1" Type="1" Reserve2=""><Section Id="专科检查 4" Type="1" Reserve2=""><Content_Text>/</Content_Text></Section><Content_Text>/</Content_Text></Section><Section Id="专科检查 2" Type="1" Reserve2=""><Content_Text>/</Content_Text></Section><Content_Text>病程 3 天以内的血常规及影像学检查（最严重的）：

/

病程 4-7 天的血常规及影像学检查（最严重的）：

/

其他检查：

</Content_Text></Section><Section Id="专科检查 3" Type="1" Reserve2=""><Content_Text>

外院

5.4 血常规：白细胞计数 4.7×10^9/L，中性粒细胞% 47%，淋巴细胞% 44.2%，血红蛋白测定 100g/L，血小板计数 138×10^9/L。

5.8 血培养：人葡萄球菌人亚型。

药敏:cefoxitin +，青霉素 R，苯唑西林 R，庆大霉素 S，环丙沙星 R，左氧氟沙星 R，莫西沙星 R，inducible clindamycin -，红霉素 R，克林霉素 R，奎奴普丁/达福普汀 S，利奈唑胺 S，万古霉素 S，四环素 S，替加环素 S，呋喃妥因 S，利福平 S，复方新诺明 R。

5.4 胸部 HRCT 平扫：右上肺后段炎症。</Content_Text></Section><NewCtrl Id="摄片时间" Type="7" Reserve2=""><Content_Text>病程其他时间</Content_Text></NewCtrl><NewCtrl Id="渗出描述" Type="7" Reserve2=""><Content_Text>边界模糊，斑片状（1分）</Content_Text></NewCtrl><NewCtrl Id="部位" Type="7" Reserve2=""><Content_Text>单叶（1分）</Content_Text></NewCtrl><NewCtrl Id="胸腔积液" Type="7" Reserve2=""><Content_Text>无（0 分）</Content_Text></NewCtrl><NewCtrl Id="脓肿" Type="7" Reserve2=""><Content_Text>无（0 分）</Content_Text></NewCtrl><NewCtrl Id="肺不张" Type="7" Reserve2=""><Content_Text>无（0分）</Content_Text></NewCtrl><NewCtrl Id="数据元73" Type="4" Reserve2=""><Content_Text /></NewCtrl><NewCtrl Id="数据元83" Type="4" Reserve2=""><Content_Text /></NewCtrl><NewCtrl Id="数据元74" Type="4" Reserve2=""><Content_Text /></NewCtrl><NewCtrl Id="数据元75" Type="4" Reserve2=""><Content_Text /></NewCtrl><NewCtrl Id="数据元76" Type="7"

Reserve2=""><Content_Text /></NewCtrl><NewCtrl Id=" 数 据 元 77" Type="4"
Reserve2=""><Content_Text /></NewCtrl><NewCtrl Id=" 数 据 元 78" Type="4"
Reserve2=""><Content_Text /></NewCtrl><NewCtrl Id=" 数 据 元 79" Type="4"
Reserve2=""><Content_Text /></NewCtrl><NewCtrl Id=" 数 据 元 85" Type="4"
Reserve2=""><Content_Text /></NewCtrl><NewCtrl Id=" 数 据 元 80" Type="4"
Reserve2=""><Content_Text /></NewCtrl><NewCtrl Id=" 数 据 元 84" Type="4"
Reserve2=""><Content_Text /></NewCtrl><NewCtrl Id=" 数 据 元 37" Type="4"
Reserve2=""><Content_Text /></NewCtrl><NewCtrl Id=" 数 据 元 38" Type="4"
Reserve2=""><Content_Text /></NewCtrl><NewCtrl Id=" 数 据 元 39" Type="4"
Reserve2=""><Content_Text /></NewCtrl><NewCtrl Id=" 数 据 元 40" Type="4"
Reserve2=""><Content_Text /></NewCtrl><NewCtrl Id=" 数 据 元 42" Type="4"
Reserve2=""><Content_Text /></NewCtrl><NewCtrl Id=" 数 据 元 43" Type="4"
Reserve2=""><Content_Text /></NewCtrl><NewCtrl Id=" 数 据 元 36" Type="4"
Reserve2=""><Content_Text /></NewCtrl><NewCtrl Id=" 数 据 元 8" Type="3"
Reserve2=""><Content_Text /></NewCtrl><NewCtrl Id=" 数 据 元 11" Type="4"
Reserve2=""><Content_Text /></NewCtrl><NewCtrl Id=" 数 据 元 21" Type="4"
Reserve2=""><Content_Text /></NewCtrl><NewCtrl Id=" 数 据 元 211" Type="4"
Reserve2=""><Content_Text /></NewCtrl><NewCtrl Id=" 数 据 元 29" Type="4"
Reserve2=""><Content_Text /></NewCtrl><NewCtrl Id=" 数 据 元 2610" Type="4"
Reserve2=""><Content_Text /></NewCtrl><NewCtrl Id=" 数 据 元 271" Type="4"
Reserve2=""><Content_Text /></NewCtrl><NewCtrl Id=" 数 据 元 2101" Type="4"
Reserve2=""><Content_Text /></NewCtrl><NewCtrl Id=" 数 据 元 14" Type="4"

Reserve2=""><Content_Text /></NewCtrl><NewCtrl Id=" 数 据 元 23" Type="4"
Reserve2=""><Content_Text /></NewCtrl><NewCtrl Id=" 数 据 元 24" Type="4"
Reserve2=""><Content_Text /></NewCtrl><NewCtrl Id=" 数 据 元 251" Type="4"
Reserve2=""><Content_Text /></NewCtrl><NewCtrl Id=" 数 据 元 51" Type="4"
Reserve2=""><Content_Text /></NewCtrl><NewCtrl Id=" 数 据 元 111" Type="4"
Reserve2=""><Content_Text /></NewCtrl><NewCtrl Id=" 数 据 元 2111" Type="4"
Reserve2=""><Content_Text /></NewCtrl><NewCtrl Id=" 数 据 元 82" Type="4"
Reserve2=""><Content_Text /></NewCtrl><NewCtrl Id=" 数 据 元 2121" Type="4"
Reserve2=""><Content_Text /></NewCtrl><NewCtrl Id=" 数 据 元 22" Type="4"
Reserve2=""><Content_Text /></NewCtrl><NewCtrl Id=" 数 据 元 9" Type="4"
Reserve2=""><Content_Text /></NewCtrl><NewCtrl Id=" 数 据 元 10" Type="4"
Reserve2=""><Content_Text /></NewCtrl><NewCtrl Id=" 数 据 元 171" Type="4"
Reserve2=""><Content_Text /></NewCtrl><NewCtrl Id=" 数 据 元 2131" Type="4"
Reserve2=""><Content_Text /></NewCtrl><NewCtrl Id=" 数 据 元 157" Type="4"
Reserve2=""><Content_Text /></NewCtrl><NewCtrl Id=" 数 据 元 2151" Type="4"
Reserve2=""><Content_Text /></NewCtrl><NewCtrl Id=" 数 据 元 2141" Type="4"
Reserve2=""><Content_Text /></NewCtrl><NewCtrl Id=" 数 据 元 16" Type="4"
Reserve2=""><Content_Text /></NewCtrl><NewCtrl Id=" 数 据 元 20" Type="4"
Reserve2=""><Content_Text /></NewCtrl><NewCtrl Id=" 数 据 元 28" Type="4"
Reserve2=""><Content_Text /></NewCtrl><NewCtrl Id=" 数 据 元 30" Type="4"
Reserve2=""><Content_Text /></NewCtrl><NewCtrl Id=" 数 据 元 2161" Type="4"
Reserve2=""><Content_Text /></NewCtrl><NewCtrl Id=" 数 据 元 2171" Type="4"

Reserve2=""><Content_Text /></NewCtrl><NewCtrl Id=" 数 据 元 2181" Type="4"
Reserve2=""><Content_Text /></NewCtrl><NewCtrl Id=" 数 据 元 2191" Type="4"
Reserve2=""><Content_Text /></NewCtrl><NewCtrl Id=" 数 据 元 2201" Type="4"
Reserve2=""><Content_Text /></NewCtrl><NewCtrl Id=" 数 据 元 224" Type="4"
Reserve2=""><Content_Text /></NewCtrl><NewCtrl Id=" 数 据 元 2231" Type="4"
Reserve2=""><Content_Text /></NewCtrl><NewCtrl Id=" 数 据 元 19" Type="4"
Reserve2=""><Content_Text /></NewCtrl><NewCtrl Id=" 数 据 元 225" Type="4"
Reserve2=""><Content_Text /></NewCtrl><NewCtrl Id=" 数 据 元 13" Type="4"
Reserve2=""><Content_Text /></NewCtrl><NewCtrl Id=" 数 据 元 41" Type="4"
Reserve2=""><Content_Text /></NewCtrl><NewCtrl Id=" 数 据 元 12" Type="4"
Reserve2=""><Content_Text /></NewCtrl><NewCtrl Id=" 数 据 元 18" Type="4"
Reserve2=""><Content_Text /></NewCtrl><NewCtrl Id=" 数 据 元 46" Type="4"
Reserve2=""><Content_Text /></NewCtrl><NewCtrl Id=" 数 据 元 31" Type="4"
Reserve2=""><Content_Text /></NewCtrl><NewCtrl Id=" 数 据 元 63" Type="4"
Reserve2=""><Content_Text /></NewCtrl><NewCtrl Id=" 数 据 元 48" Type="4"
Reserve2=""><Content_Text /></NewCtrl><NewCtrl Id=" 数 据 元 71" Type="4"
Reserve2=""><Content_Text /></NewCtrl><NewCtrl Id=" 数 据 元 269" Type="3"
Reserve2=""><Content_Text>11. 初 步 诊 断 ： </Content_Text></NewCtrl><Section
Id="section130401212425" Type="1" Reserve2=""><Section Id="Custom_InitialDiagnosis"
Type="1" Reserve2=""><NewCtrl Id="Admissiondiagnosis" Type="3"
Reserve2=""><Content_Text>1.社区获得性肺炎，非重症 2.癫痫 3.先天性喉软骨软化病 4.
生长发育迟缓 </Content_Text></NewCtrl><Content_Text>1.社区获得性肺炎，非重症 2.

癫痫 3.先天性喉软骨软化病 4.生长发育迟缓 </Content_Text></Section><Content_Text>

1.社区获得性肺炎，非重症 2.癫痫 3.先天性喉软骨软化病 4.生长发育迟缓 </Content_Text></Section><Section Id="Section_130105135944723" Type="1" Reserve2=""><NewCtrl Id="Metadata_RecordDoctorName" Type="3" Reserve2=""><Content_Text>袁姝华</Content_Text></NewCtrl><NewCtrl Id="数据元 35" Type="11" Reserve2=""><Content_Text>2020-05-12</Content_Text></NewCtrl><Content_Text>记录医师:　　　　签名:

记录时间: 2020-05-12 时间:

主治医师签名:

日期:　　　年　　月　　日　　时间

</Content_Text></Section></DocObjContent>

Supplementary file2: the SQL code for extracting the main items of the SEMRs

```
SELECT p.PatientID, CureNo, emr_doc.BedCode, emr_doc.PatientName, p.BirthDay as '出生日期', Age, SexDisplay, p.AdmissionDate as '入院时间', p.DischargeDate as '出院时间',[dbo].[f_PatientDiagnosisInfo](emr_doc.CureNo) as '诊断',
'身高' = [dbo].[EXT_ISNULL](xmlIsland.query(N'//NewCtrl[@Id="身长高值"]/Content_Text[last()]').value('(/)[1]', 'nvarchar(max)'),xmlIsland.query(N'//NewCtrl[@Id="身长高值1"]/Content_Text[last()]').value('(/)[1]', 'nvarchar(max)'),''),
'体重' = [dbo].[EXT_ISNULL](xmlIsland.query(N'//NewCtrl[@Id="体重值"]/Content_Text[last()]').value('(/)[1]', 'nvarchar(max)'),xmlIsland.query(N'//NewCtrl[@Id="体重值1"]/Content_Text[last()]').value('(/)[1]', 'nvarchar(max)'),''),
'主诉' = xmlIsland.query(N'//Section[@Id="Section_ChiefComplaint"]/Content_Text[last()]').value('(/)[1]', 'nvarchar(max)'),
'发热'= [dbo].[EXT_ISNULL](xmlIsland.query(N'//NewCtrl[@Id="有无发热"]/Content_Text[last()]').value('(/)[1]', 'nvarchar(max)'),xmlIsland.query(N'//NewCtrl[@Id="有无发热1"]/Content_Text[last()]').value('(/)[1]', 'nvarchar(max)'),''),
'发热时间'= [dbo].[EXT_ISNULL](xmlIsland.query(N'//NewCtrl[@Id="发热时间"]/Content_Text[last()]').value('(/)[1]', 'nvarchar(max)'),xmlIsland.query(N'//NewCtrl[@Id="发热时间
```

```
1"]/Content_Text[last()]').value('(/)[1]', 'nvarchar(max)'),''),

'发热时长单位'= [dbo].[EXT_ISNULL](xmlIsland.query(N'//NewCtrl[@Id="发热时长单

位"]/Content_Text[last()]').value('(/)[1]',

'nvarchar(max)'),xmlIsland.query(N'//NewCtrl[@Id="发热发热时长单位

1"]/Content_Text[last()]').value('(/)[1]', 'nvarchar(max)'),''),

'热峰' = [dbo].[EXT_ISNULL](xmlIsland.query(N'//NewCtrl[@Id="热峰

"]/Content_Text[last()]').value('(/)[1]',

'nvarchar(max)'),xmlIsland.query(N'//NewCtrl[@Id="热峰

1"]/Content_Text[last()]').value('(/)[1]', 'nvarchar(max)'),''),

'咳嗽' = xmlIsland.query(N'//NewCtrl[@Id="咳嗽时相

"]/Content_Text[last()]').value('(/)[1]', 'nvarchar(max)'),

'咳嗽时间' = [dbo].[EXT_ISNULL](xmlIsland.query(N'//NewCtrl[@Id="咳嗽时间

"]/Content_Text[last()]').value('(/)[1]',

'nvarchar(max)'),xmlIsland.query(N'//NewCtrl[@Id="咳嗽时间

1"]/Content_Text[last()]').value('(/)[1]', 'nvarchar(max)'),''),

'咳嗽时长单位' = [dbo].[EXT_ISNULL](xmlIsland.query(N'//NewCtrl[@Id="咳嗽时长单

位"]/Content_Text[last()]').value('(/)[1]',

'nvarchar(max)'),xmlIsland.query(N'//NewCtrl[@Id="咳嗽时长单位

1"]/Content_Text[last()]').value('(/)[1]', 'nvarchar(max)'),''),

'是否咳痰' = [dbo].[EXT_ISNULL](xmlIsland.query(N'//NewCtrl[@Id="是否咳痰

"]/Content_Text[last()]').value('(/)[1]',

'nvarchar(max)'),xmlIsland.query(N'//NewCtrl[@Id="是否咳痰
```

```
1"]/Content_Text[last()]').value('(/)[1]', 'nvarchar(max)'),''),
'咳嗽性质' = [dbo].[EXT_ISNULL](xmlIsland.query(N'//NewCtrl[@Id="咳嗽性质
"]/Content_Text[last()]').value('(/)[1]',
'nvarchar(max)'),xmlIsland.query(N'//NewCtrl[@Id="咳嗽性质
1"]/Content_Text[last()]').value('(/)[1]', 'nvarchar(max)'),''),
'咳嗽严重程度' = [dbo].[EXT_ISNULL](xmlIsland.query(N'//NewCtrl[@Id="咳嗽严重程
度"]/Content_Text[last()]').value('(/)[1]',
'nvarchar(max)'),xmlIsland.query(N'//NewCtrl[@Id="咳嗽严重程度
1"]/Content_Text[last()]').value('(/)[1]', 'nvarchar(max)'),''),
'喘息情况' = [dbo].[EXT_ISNULL](xmlIsland.query(N'//NewCtrl[@Id="喘息情况
"]/Content_Text[last()]').value('(/)[1]',
'nvarchar(max)'),xmlIsland.query(N'//NewCtrl[@Id="喘息情况
1"]/Content_Text[last()]').value('(/)[1]', 'nvarchar(max)'),''),
'喘息时间' = [dbo].[EXT_ISNULL](xmlIsland.query(N'//NewCtrl[@Id="喘息时间
"]/Content_Text[last()]').value('(/)[1]',
'nvarchar(max)'),xmlIsland.query(N'//NewCtrl[@Id="喘息时间
1"]/Content_Text[last()]').value('(/)[1]', 'nvarchar(max)'),''),
'喘息时长单位' = [dbo].[EXT_ISNULL](xmlIsland.query(N'//NewCtrl[@Id="喘息时长单
位"]/Content_Text[last()]').value('(/)[1]',
'nvarchar(max)'),xmlIsland.query(N'//NewCtrl[@Id="喘息时长单位
1"]/Content_Text[last()]').value('(/)[1]', 'nvarchar(max)'),''),
'有无异物吸入' = [dbo].[EXT_ISNULL](xmlIsland.query(N'//NewCtrl[@Id="有无异物吸
```

```
入"]/Content_Text[last()]').value('(/)[1]',
'nvarchar(max)'),xmlIsland.query(N'//NewCtrl[@Id="有无异物吸入
1"]/Content_Text[last()]').value('(/)[1]', 'nvarchar(max)'),''),
'呼吸伴随症状' = [dbo].[EXT_ISNULL](xmlIsland.query(N'//NewCtrl[@Id="呼吸伴随症
状"]/Content_Text[last()]').value('(/)[1]',
'nvarchar(max)'),xmlIsland.query(N'//NewCtrl[@Id="呼吸伴随症状
1"]/Content_Text[last()]').value('(/)[1]', 'nvarchar(max)'),''),
'精神情况' = [dbo].[EXT_ISNULL](xmlIsland.query(N'//NewCtrl[@Id="精神情况
"]/Content_Text[last()]').value('(/)[1]',
'nvarchar(max)'),xmlIsland.query(N'//NewCtrl[@Id="精神情况
1"]/Content_Text[last()]').value('(/)[1]', 'nvarchar(max)'),''),
'胃纳' = [dbo].[EXT_ISNULL](xmlIsland.query(N'//NewCtrl[@Id="胃纳
"]/Content_Text[last()]').value('(/)[1]',
'nvarchar(max)'),xmlIsland.query(N'//NewCtrl[@Id="胃纳
1"]/Content_Text[last()]').value('(/)[1]', 'nvarchar(max)'),''),
'发热伴随症状' = [dbo].[EXT_ISNULL](xmlIsland.query(N'//NewCtrl[@Id="喘息时长单
位"]/Content_Text[last()]').value('(/)[1]',
'nvarchar(max)'),xmlIsland.query(N'//NewCtrl[@Id="喘息时长单位
1"]/Content_Text[last()]').value('(/)[1]', 'nvarchar(max)'),''),
'头面部症状' = [dbo].[EXT_ISNULL](xmlIsland.query(N'//NewCtrl[@Id="头面部症状
"]/Content_Text[last()]').value('(/)[1]',
'nvarchar(max)'),xmlIsland.query(N'//NewCtrl[@Id="头面部症状
```

```
1"]/Content_Text[last()]').value('(/)[1]', 'nvarchar(max)'),''),
'上气道症状' = [dbo].[EXT_ISNULL](xmlIsland.query(N'//NewCtrl[@Id="上气道症状
"]/Content_Text[last()]').value('(/)[1]',
'nvarchar(max)'),xmlIsland.query(N'//NewCtrl[@Id="上气道症状
1"]/Content_Text[last()]').value('(/)[1]', 'nvarchar(max)'),''),
'皮肤' = [dbo].[EXT_ISNULL](xmlIsland.query(N'//NewCtrl[@Id="皮肤
"]/Content_Text[last()]').value('(/)[1]',
'nvarchar(max)'),xmlIsland.query(N'//NewCtrl[@Id="皮肤
1"]/Content_Text[last()]').value('(/)[1]', 'nvarchar(max)'),''),
'神经系统' = [dbo].[EXT_ISNULL](xmlIsland.query(N'//NewCtrl[@Id="神经系统
"]/Content_Text[last()]').value('(/)[1]',
'nvarchar(max)'),xmlIsland.query(N'//NewCtrl[@Id="神经系统
1"]/Content_Text[last()]').value('(/)[1]', 'nvarchar(max)'),''),
'泌尿系统' = [dbo].[EXT_ISNULL](xmlIsland.query(N'//NewCtrl[@Id="泌尿系统
"]/Content_Text[last()]').value('(/)[1]',
'nvarchar(max)'),xmlIsland.query(N'//NewCtrl[@Id="泌尿系统
1"]/Content_Text[last()]').value('(/)[1]', 'nvarchar(max)'),''),
'肺链疫苗接种史' = [dbo].[EXT_ISNULL](xmlIsland.query(N'//NewCtrl[@Id="肺链疫苗
接种史"]/Content_Text[last()]').value('(/)[1]',
'nvarchar(max)'),xmlIsland.query(N'//NewCtrl[@Id="肺链疫苗接种史
1"]/Content_Text[last()]').value('(/)[1]', 'nvarchar(max)'),''),
'药物食物过敏史' = [dbo].[EXT_ISNULL](xmlIsland.query(N'//NewCtrl[@Id="药物食物
```

```
过敏史"]/Content_Text[last()]').value('(/)[1]',
'nvarchar(max)'),xmlIsland.query(N'//NewCtrl[@Id="药物食物过敏史
1"]/Content_Text[last()]').value('(/)[1]', 'nvarchar(max)'),''),
'湿疹史' = [dbo].[EXT_ISNULL](xmlIsland.query(N'//NewCtrl[@Id="湿疹史
"]/Content_Text[last()]').value('(/)[1]',
'nvarchar(max)'),xmlIsland.query(N'//NewCtrl[@Id="湿疹史
1"]/Content_Text[last()]').value('(/)[1]', 'nvarchar(max)'),''),
'牛奶蛋白过敏' = [dbo].[EXT_ISNULL](xmlIsland.query(N'//NewCtrl[@Id="牛奶蛋白过
敏"]/Content_Text[last()]').value('(/)[1]',
'nvarchar(max)'),xmlIsland.query(N'//NewCtrl[@Id="牛奶蛋白过敏
1"]/Content_Text[last()]').value('(/)[1]', 'nvarchar(max)'),''),
'过敏性鼻炎' = [dbo].[EXT_ISNULL](xmlIsland.query(N'//NewCtrl[@Id="过敏性鼻炎
"]/Content_Text[last()]').value('(/)[1]',
'nvarchar(max)'),xmlIsland.query(N'//NewCtrl[@Id="过敏性鼻炎
1"]/Content_Text[last()]').value('(/)[1]', 'nvarchar(max)'),''),
'呼吸系统回顾' = [dbo].[EXT_ISNULL](xmlIsland.query(N'//NewCtrl[@Id="呼吸系统回
顾"]/Content_Text[last()]').value('(/)[1]',
'nvarchar(max)'),xmlIsland.query(N'//NewCtrl[@Id="呼吸系统回顾
1"]/Content_Text[last()]').value('(/)[1]', 'nvarchar(max)'),''),
'反复呼吸道感染' = [dbo].[EXT_ISNULL](xmlIsland.query(N'//NewCtrl[@Id="反复呼吸
道感染"]/Content_Text[last()]').value('(/)[1]',
'nvarchar(max)'),xmlIsland.query(N'//NewCtrl[@Id="反复呼吸道感染
```

```
1"]/Content_Text[last()]').value('(/)[1]', 'nvarchar(max)'),''),
'既往喘息史' = [dbo].[EXT_ISNULL](xmlIsland.query(N'//NewCtrl[@Id="既往喘息史
"]/Content_Text[last()]').value('(/)[1]',
'nvarchar(max)'),xmlIsland.query(N'//NewCtrl[@Id="既往喘息史
1"]/Content_Text[last()]').value('(/)[1]', 'nvarchar(max)'),''),
'上呼吸道感染次数' = [dbo].[EXT_ISNULL](xmlIsland.query(N'//NewCtrl[@Id="上呼吸
道感染次数"]/Content_Text[last()]').value('(/)[1]',
'nvarchar(max)'),xmlIsland.query(N'//NewCtrl[@Id="上呼吸道感染次数
1"]/Content_Text[last()]').value('(/)[1]', 'nvarchar(max)'),''),
'下呼吸道感染次数' = [dbo].[EXT_ISNULL](xmlIsland.query(N'//NewCtrl[@Id="下呼吸
道感染次数"]/Content_Text[last()]').value('(/)[1]',
'nvarchar(max)'),xmlIsland.query(N'//NewCtrl[@Id="下呼吸道感染次数
1"]/Content_Text[last()]').value('(/)[1]', 'nvarchar(max)'),''),
'住院次数' = [dbo].[EXT_ISNULL](xmlIsland.query(N'//NewCtrl[@Id="住院次数
"]/Content_Text[last()]').value('(/)[1]',
'nvarchar(max)'),xmlIsland.query(N'//NewCtrl[@Id="住院次数
1"]/Content_Text[last()]').value('(/)[1]', 'nvarchar(max)'),''),
'吸氧次数' = [dbo].[EXT_ISNULL](xmlIsland.query(N'//NewCtrl[@Id="吸氧次数
"]/Content_Text[last()]').value('(/)[1]',
'nvarchar(max)'),xmlIsland.query(N'//NewCtrl[@Id="吸氧次数
1"]/Content_Text[last()]').value('(/)[1]', 'nvarchar(max)'),''),
'ICU 次数' = [dbo].[EXT_ISNULL](xmlIsland.query(N'//NewCtrl[@Id="ICU 次数
```

```
"]/Content_Text[last()]').value('(/)[1]',
'nvarchar(max)'),xmlIsland.query(N'//NewCtrl[@Id="ICU 次数
1"]/Content_Text[last()]').value('(/)[1]', 'nvarchar(max)'),''),
'有无呼吸系统基础疾病' = [dbo].[EXT_ISNULL](xmlIsland.query(N'//NewCtrl[@Id="有
无呼吸系统基础疾病"]/Content_Text[last()]').value('(/)[1]',
'nvarchar(max)'),xmlIsland.query(N'//NewCtrl[@Id="有无呼吸系统基础疾病
1"]/Content_Text[last()]').value('(/)[1]', 'nvarchar(max)'),''),
'呼吸系统严重疾病' = [dbo].[EXT_ISNULL](xmlIsland.query(N'//NewCtrl[@Id="呼吸系
统严重疾病"]/Content_Text[last()]').value('(/)[1]',
'nvarchar(max)'),xmlIsland.query(N'//NewCtrl[@Id="呼吸系统严重疾病
1"]/Content_Text[last()]').value('(/)[1]', 'nvarchar(max)'),''),
'有无其他基础疾病' = [dbo].[EXT_ISNULL](xmlIsland.query(N'//NewCtrl[@Id="既往喘
息史"]/Content_Text[last()]').value('(/)[1]',
'nvarchar(max)'),xmlIsland.query(N'//NewCtrl[@Id="既往喘息史
1"]/Content_Text[last()]').value('(/)[1]', 'nvarchar(max)'),''),
'首次喘息月龄' = [dbo].[EXT_ISNULL](xmlIsland.query(N'//NewCtrl[@Id="首次喘息月
龄"]/Content_Text[last()]').value('(/)[1]',
'nvarchar(max)'),xmlIsland.query(N'//NewCtrl[@Id="既往喘息史
1"]/Content_Text[last()]').value('(/)[1]', 'nvarchar(max)'),''),
'喘息次数 2 年内' = [dbo].[EXT_ISNULL](xmlIsland.query(N'//NewCtrl[@Id="喘息次数
2 年内"]/Content_Text[last()]').value('(/)[1]',
'nvarchar(max)'),xmlIsland.query(N'//NewCtrl[@Id="既往喘息史
```

```
1"]/Content_Text[last()]').value('(/)[1]', 'nvarchar(max)'),''),

'喘息次数 1 年内' = [dbo].[EXT_ISNULL](xmlIsland.query(N'//NewCtrl[@Id="喘息次数

1 年内"]/Content_Text[last()]').value('(/)[1]',

'nvarchar(max)'),xmlIsland.query(N'//NewCtrl[@Id="喘息次数 1 年内

1"]/Content_Text[last()]').value('(/)[1]', 'nvarchar(max)'),''),

'首次喘息月龄' = [dbo].[EXT_ISNULL](xmlIsland.query(N'//NewCtrl[@Id="首次喘息月

龄"]/Content_Text[last()]').value('(/)[1]',

'nvarchar(max)'),xmlIsland.query(N'//NewCtrl[@Id="首次喘息月龄

1"]/Content_Text[last()]').value('(/)[1]', 'nvarchar(max)'),''),

'喘息次数' = [dbo].[EXT_ISNULL](xmlIsland.query(N'//NewCtrl[@Id="喘息次数

"]/Content_Text[last()]').value('(/)[1]',

'nvarchar(max)'),xmlIsland.query(N'//NewCtrl[@Id="喘息次数

1"]/Content_Text[last()]').value('(/)[1]', 'nvarchar(max)'),''),

'诱发因素' = [dbo].[EXT_ISNULL](xmlIsland.query(N'//NewCtrl[@Id="诱发因素

"]/Content_Text[last()]').value('(/)[1]',

'nvarchar(max)'),xmlIsland.query(N'//NewCtrl[@Id="诱发因素

1"]/Content_Text[last()]').value('(/)[1]', 'nvarchar(max)'),''),

'过敏诱发喘息' = [dbo].[EXT_ISNULL](xmlIsland.query(N'//NewCtrl[@Id="过敏诱发喘

息"]/Content_Text[last()]').value('(/)[1]',

'nvarchar(max)'),xmlIsland.query(N'//NewCtrl[@Id="过敏诱发喘息

1"]/Content_Text[last()]').value('(/)[1]', 'nvarchar(max)'),''),

'舒张剂治疗效果' = [dbo].[EXT_ISNULL](xmlIsland.query(N'//NewCtrl[@Id="舒张剂治
```

```
疗效果"]/Content_Text[last()]').value('(/)[1]',
'nvarchar(max)'),xmlIsland.query(N'//NewCtrl[@Id="舒张剂治疗效果
1"]/Content_Text[last()]').value('(/)[1]', 'nvarchar(max)'),''),
'血清总 IgE' = [dbo].[EXT_ISNULL](xmlIsland.query(N'//NewCtrl[@Id="血清总
IgE"]/Content_Text[last()]').value('(/)[1]',
'nvarchar(max)'),xmlIsland.query(N'//NewCtrl[@Id="血清总
IgE1"]/Content_Text[last()]').value('(/)[1]', 'nvarchar(max)'),''),
'皮肤点刺' = [dbo].[EXT_ISNULL](xmlIsland.query(N'//NewCtrl[@Id="皮肤点刺
"]/Content_Text[last()]').value('(/)[1]',
'nvarchar(max)'),xmlIsland.query(N'//NewCtrl[@Id="皮肤点刺
1"]/Content_Text[last()]').value('(/)[1]', 'nvarchar(max)'),''),
'过敏原' = [dbo].[EXT_ISNULL](xmlIsland.query(N'//NewCtrl[@Id="过敏原
"]/Content_Text[last()]').value('(/)[1]',
'nvarchar(max)'),xmlIsland.query(N'//NewCtrl[@Id="过敏原
1"]/Content_Text[last()]').value('(/)[1]', 'nvarchar(max)'),''),
'治疗哮喘日期' = [dbo].[EXT_ISNULL](xmlIsland.query(N'//NewCtrl[@Id="治疗哮喘日
期"]/Content_Text[last()]').value('(/)[1]',
'nvarchar(max)'),xmlIsland.query(N'//NewCtrl[@Id="治疗哮喘日期
1"]/Content_Text[last()]').value('(/)[1]', 'nvarchar(max)'),''),
'诊断哮喘' = [dbo].[EXT_ISNULL](xmlIsland.query(N'//NewCtrl[@Id="诊断哮喘
"]/Content_Text[last()]').value('(/)[1]',
'nvarchar(max)'),xmlIsland.query(N'//NewCtrl[@Id="诊断哮喘
```

```
1"]/Content_Text[last()]').value('(/)[1]', 'nvarchar(max)'),''),

'哮喘用药' = [dbo].[EXT_ISNULL](xmlIsland.query(N'//NewCtrl[@Id="哮喘用药

"]/Content_Text[last()]').value('(/)[1]',

'nvarchar(max)'),xmlIsland.query(N'//NewCtrl[@Id="哮喘用药

1"]/Content_Text[last()]').value('(/)[1]', 'nvarchar(max)'),''),

'依从性' = [dbo].[EXT_ISNULL](xmlIsland.query(N'//NewCtrl[@Id="依从性

"]/Content_Text[last()]').value('(/)[1]',

'nvarchar(max)'),xmlIsland.query(N'//NewCtrl[@Id="依从性

1"]/Content_Text[last()]').value('(/)[1]', 'nvarchar(max)'),''),

'哮喘控制情况' = [dbo].[EXT_ISNULL](xmlIsland.query(N'//NewCtrl[@Id="哮喘控制情

况"]/Content_Text[last()]').value('(/)[1]',

'nvarchar(max)'),xmlIsland.query(N'//NewCtrl[@Id="哮喘控制情况

1"]/Content_Text[last()]').value('(/)[1]', 'nvarchar(max)'),''),

'生产情况' = [dbo].[EXT_ISNULL](xmlIsland.query(N'//NewCtrl[@Id="生产情况

"]/Content_Text[last()]').value('(/)[1]',

'nvarchar(max)'),xmlIsland.query(N'//NewCtrl[@Id="生产情况

1"]/Content_Text[last()]').value('(/)[1]', 'nvarchar(max)'),''),

'出生体重' = [dbo].[EXT_ISNULL](xmlIsland.query(N'//NewCtrl[@Id="出生体重

"]/Content_Text[last()]').value('(/)[1]',

'nvarchar(max)'),xmlIsland.query(N'//NewCtrl[@Id="出生体重

1"]/Content_Text[last()]').value('(/)[1]', 'nvarchar(max)'),''),

'喂养方式' = [dbo].[EXT_ISNULL](xmlIsland.query(N'//NewCtrl[@Id="喂养方式
```

```
"]/Content_Text[last()]').value('(/)[1]',
'nvarchar(max)'),xmlIsland.query(N'//NewCtrl[@Id="喂养方式
1"]/Content_Text[last()]').value('(/)[1]', 'nvarchar(max)'),''),
'添加辅食情况' = [dbo].[EXT_ISNULL](xmlIsland.query(N'//NewCtrl[@Id="添加辅食情
况"]/Content_Text[last()]').value('(/)[1]',
'nvarchar(max)'),xmlIsland.query(N'//NewCtrl[@Id="添加辅食情况
1"]/Content_Text[last()]').value('(/)[1]', 'nvarchar(max)'),''),
'生长发育' = [dbo].[EXT_ISNULL](xmlIsland.query(N'//NewCtrl[@Id="生长发育
"]/Content_Text[last()]').value('(/)[1]',
'nvarchar(max)'),xmlIsland.query(N'//NewCtrl[@Id="生长发育
1"]/Content_Text[last()]').value('(/)[1]', 'nvarchar(max)'),''),
'父亲年龄' = [dbo].[EXT_ISNULL](xmlIsland.query(N'//NewCtrl[@Id="父亲年龄
"]/Content_Text[last()]').value('(/)[1]',
'nvarchar(max)'),xmlIsland.query(N'//NewCtrl[@Id="父亲年龄
1"]/Content_Text[last()]').value('(/)[1]', 'nvarchar(max)'),''),
'父亲职业' = [dbo].[EXT_ISNULL](xmlIsland.query(N'//NewCtrl[@Id="父亲职业
"]/Content_Text[last()]').value('(/)[1]',
'nvarchar(max)'),xmlIsland.query(N'//NewCtrl[@Id="父亲职业
1"]/Content_Text[last()]').value('(/)[1]', 'nvarchar(max)'),''),
'母亲年龄' = [dbo].[EXT_ISNULL](xmlIsland.query(N'//NewCtrl[@Id="母亲年龄
"]/Content_Text[last()]').value('(/)[1]',
'nvarchar(max)'),xmlIsland.query(N'//NewCtrl[@Id="母亲年龄
```

```
1"]/Content_Text[last()]').value('(/)[1]', 'nvarchar(max)'),''),
'母亲职业' = [dbo].[EXT_ISNULL](xmlIsland.query(N'//NewCtrl[@Id="母亲职业
"]/Content_Text[last()]').value('(/)[1]',
'nvarchar(max)'),xmlIsland.query(N'//NewCtrl[@Id="母亲职业
1"]/Content_Text[last()]').value('(/)[1]', 'nvarchar(max)'),''),
'父亲健康情况' = [dbo].[EXT_ISNULL](xmlIsland.query(N'//NewCtrl[@Id="父亲健康情
况"]/Content_Text[last()]').value('(/)[1]',
'nvarchar(max)'),xmlIsland.query(N'//NewCtrl[@Id="父亲健康情况
1"]/Content_Text[last()]').value('(/)[1]', 'nvarchar(max)'),''),
'母亲健康情况' = [dbo].[EXT_ISNULL](xmlIsland.query(N'//NewCtrl[@Id="母亲健康情
况"]/Content_Text[last()]').value('(/)[1]',
'nvarchar(max)'),xmlIsland.query(N'//NewCtrl[@Id="母亲健康情况
1"]/Content_Text[last()]').value('(/)[1]', 'nvarchar(max)'),''),
'家庭成员健康情况' = [dbo].[EXT_ISNULL](xmlIsland.query(N'//NewCtrl[@Id="家庭成
员健康情况"]/Content_Text[last()]').value('(/)[1]',
'nvarchar(max)'),xmlIsland.query(N'//NewCtrl[@Id="家庭成员健康情况
1"]/Content_Text[last()]').value('(/)[1]', 'nvarchar(max)'),''),
'吸烟暴露' = [dbo].[EXT_ISNULL](xmlIsland.query(N'//NewCtrl[@Id="吸烟暴露
"]/Content_Text[last()]').value('(/)[1]',
'nvarchar(max)'),xmlIsland.query(N'//NewCtrl[@Id="吸烟暴露
1"]/Content_Text[last()]').value('(/)[1]', 'nvarchar(max)'),''),
'体温测量方式' = [dbo].[EXT_ISNULL](xmlIsland.query(N'//NewCtrl[@Id="体温测量方
```

```
式"]/Content_Text[last()]').value('(/)[1]',

'nvarchar(max)'),xmlIsland.query(N'//NewCtrl[@Id="体温测量方式

1"]/Content_Text[last()]').value('(/)[1]', 'nvarchar(max)'),''),

'体温值' = [dbo].[EXT_ISNULL](xmlIsland.query(N'//NewCtrl[@Id="体温值

"]/Content_Text[last()]').value('(/)[1]',

'nvarchar(max)'),xmlIsland.query(N'//NewCtrl[@Id="体温值

1"]/Content_Text[last()]').value('(/)[1]', 'nvarchar(max)'),''),

'脉搏次数' = [dbo].[EXT_ISNULL](xmlIsland.query(N'//NewCtrl[@Id="脉搏次数

"]/Content_Text[last()]').value('(/)[1]',

'nvarchar(max)'),xmlIsland.query(N'//NewCtrl[@Id="脉搏次数

1"]/Content_Text[last()]').value('(/)[1]', 'nvarchar(max)'),''),

'呼吸次数' = [dbo].[EXT_ISNULL](xmlIsland.query(N'//NewCtrl[@Id="呼吸次数

"]/Content_Text[last()]').value('(/)[1]',

'nvarchar(max)'),xmlIsland.query(N'//NewCtrl[@Id="呼吸次数

1"]/Content_Text[last()]').value('(/)[1]', 'nvarchar(max)'),''),

'收缩压' = [dbo].[EXT_ISNULL](xmlIsland.query(N'//NewCtrl[@Id="收缩压

"]/Content_Text[last()]').value('(/)[1]',

'nvarchar(max)'),xmlIsland.query(N'//NewCtrl[@Id="收缩压

1"]/Content_Text[last()]').value('(/)[1]', 'nvarchar(max)'),''),

'舒张压' = [dbo].[EXT_ISNULL](xmlIsland.query(N'//NewCtrl[@Id="舒张压

"]/Content_Text[last()]').value('(/)[1]',

'nvarchar(max)'),xmlIsland.query(N'//NewCtrl[@Id="舒张压
```

```
1"]/Content_Text[last()]').value('(/)[1]', 'nvarchar(max)'),''),

'身长高值' = [dbo].[EXT_ISNULL](xmlIsland.query(N'//NewCtrl[@Id="身长高值

"]/Content_Text[last()]').value('(/)[1]',

'nvarchar(max)'),xmlIsland.query(N'//NewCtrl[@Id="身长高值

1"]/Content_Text[last()]').value('(/)[1]', 'nvarchar(max)'),''),

'体重值' = [dbo].[EXT_ISNULL](xmlIsland.query(N'//NewCtrl[@Id="体重值

"]/Content_Text[last()]').value('(/)[1]',

'nvarchar(max)'),xmlIsland.query(N'//NewCtrl[@Id="体重值

1"]/Content_Text[last()]').value('(/)[1]', 'nvarchar(max)'),''),

'未吸氧下氧合' = [dbo].[EXT_ISNULL](xmlIsland.query(N'//NewCtrl[@Id="未吸氧下氧

合"]/Content_Text[last()]').value('(/)[1]',

'nvarchar(max)'),xmlIsland.query(N'//NewCtrl[@Id="未吸氧下氧合

1"]/Content_Text[last()]').value('(/)[1]', 'nvarchar(max)'),''),

'吸氧方式' = [dbo].[EXT_ISNULL](xmlIsland.query(N'//NewCtrl[@Id="吸氧方式

"]/Content_Text[last()]').value('(/)[1]',

'nvarchar(max)'),xmlIsland.query(N'//NewCtrl[@Id="吸氧方式

1"]/Content_Text[last()]').value('(/)[1]', 'nvarchar(max)'),''),

'吸氧后氧合值' = [dbo].[EXT_ISNULL](xmlIsland.query(N'//NewCtrl[@Id="吸氧后氧合

值"]/Content_Text[last()]').value('(/)[1]',

'nvarchar(max)'),xmlIsland.query(N'//NewCtrl[@Id="吸氧后氧合值

1"]/Content_Text[last()]').value('(/)[1]', 'nvarchar(max)'),''),

'胸廓畸形' = [dbo].[EXT_ISNULL](xmlIsland.query(N'//NewCtrl[@Id="胸廓畸形
```

```
"]/Content_Text[last()]').value('(/)[1]',
'nvarchar(max)'),xmlIsland.query(N'//NewCtrl[@Id="胸廓畸形
1"]/Content_Text[last()]').value('(/)[1]', 'nvarchar(max)'),''),
'胸廓' = [dbo].[EXT_ISNULL](xmlIsland.query(N'//NewCtrl[@Id="胸廓
"]/Content_Text[last()]').value('(/)[1]',
'nvarchar(max)'),xmlIsland.query(N'//NewCtrl[@Id="胸廓
1"]/Content_Text[last()]').value('(/)[1]', 'nvarchar(max)'),''),
'肺望诊情况' = [dbo].[EXT_ISNULL](xmlIsland.query(N'//NewCtrl[@Id="肺望诊情况
"]/Content_Text[last()]').value('(/)[1]',
'nvarchar(max)'),xmlIsland.query(N'//NewCtrl[@Id="肺望诊情况
1"]/Content_Text[last()]').value('(/)[1]', 'nvarchar(max)'),''),
'有无呼吸困难' = [dbo].[EXT_ISNULL](xmlIsland.query(N'//NewCtrl[@Id="有无呼吸困
难"]/Content_Text[last()]').value('(/)[1]',
'nvarchar(max)'),xmlIsland.query(N'//NewCtrl[@Id="有无呼吸困难
1"]/Content_Text[last()]').value('(/)[1]', 'nvarchar(max)'),''),
'呼吸困难表现' = [dbo].[EXT_ISNULL](xmlIsland.query(N'//NewCtrl[@Id="呼吸困难表
现"]/Content_Text[last()]').value('(/)[1]',
'nvarchar(max)'),xmlIsland.query(N'//NewCtrl[@Id="呼吸困难表现
1"]/Content_Text[last()]').value('(/)[1]', 'nvarchar(max)'),''),
'有无呼吸增快' = [dbo].[EXT_ISNULL](xmlIsland.query(N'//NewCtrl[@Id="有无呼吸增
快"]/Content_Text[last()]').value('(/)[1]',
'nvarchar(max)'),xmlIsland.query(N'//NewCtrl[@Id="有无呼吸增快
```

```
1"]/Content_Text[last()]').value('(/)[1]', 'nvarchar(max)'),''),

'呼吸音' = [dbo].[EXT_ISNULL](xmlIsland.query(N'//NewCtrl[@Id="呼吸音

"]/Content_Text[last()]').value('(/)[1]',

'nvarchar(max)'),xmlIsland.query(N'//NewCtrl[@Id="呼吸音

1"]/Content_Text[last()]').value('(/)[1]', 'nvarchar(max)'),''),

'湿罗音部位' = [dbo].[EXT_ISNULL](xmlIsland.query(N'//NewCtrl[@Id="湿罗音部位

"]/Content_Text[last()]').value('(/)[1]',

'nvarchar(max)'),xmlIsland.query(N'//NewCtrl[@Id="湿罗音部位

1"]/Content_Text[last()]').value('(/)[1]', 'nvarchar(max)'),''),

'是否有湿罗音' = [dbo].[EXT_ISNULL](xmlIsland.query(N'//NewCtrl[@Id="是否有湿罗

音"]/Content_Text[last()]').value('(/)[1]',

'nvarchar(max)'),xmlIsland.query(N'//NewCtrl[@Id="是否有湿罗音

1"]/Content_Text[last()]').value('(/)[1]', 'nvarchar(max)'),''),

'湿罗音种类' = [dbo].[EXT_ISNULL](xmlIsland.query(N'//NewCtrl[@Id="湿罗音种类

"]/Content_Text[last()]').value('(/)[1]',

'nvarchar(max)'),xmlIsland.query(N'//NewCtrl[@Id="湿罗音种类

1"]/Content_Text[last()]').value('(/)[1]', 'nvarchar(max)'),''),

'干啰音部位' = [dbo].[EXT_ISNULL](xmlIsland.query(N'//NewCtrl[@Id="干啰音部位

"]/Content_Text[last()]').value('(/)[1]',

'nvarchar(max)'),xmlIsland.query(N'//NewCtrl[@Id="干啰音部位

1"]/Content_Text[last()]').value('(/)[1]', 'nvarchar(max)'),''),

'是否有哮鸣音' = [dbo].[EXT_ISNULL](xmlIsland.query(N'//NewCtrl[@Id="是否有哮鸣
```

音"]/Content_Text[last()]').value('(/)[1]',

'nvarchar(max)'),xmlIsland.query(N'//NewCtrl[@Id="是否有哮鸣音

1"]/Content_Text[last()]').value('(/)[1]', 'nvarchar(max)'),''),

'哮鸣音' = [dbo].[EXT_ISNULL](xmlIsland.query(N'//NewCtrl[@Id="哮鸣音

"]/Content_Text[last()]').value('(/)[1]',

'nvarchar(max)'),xmlIsland.query(N'//NewCtrl[@Id="哮鸣音

1"]/Content_Text[last()]').value('(/)[1]', 'nvarchar(max)'),''),

'肺炎评分' = [dbo].[EXT_ISNULL](xmlIsland.query(N'//NewCtrl[@Id="肺炎评分

"]/Content_Text[last()]').value('(/)[1]',

'nvarchar(max)'),xmlIsland.query(N'//NewCtrl[@Id="肺炎评分

1"]/Content_Text[last()]').value('(/)[1]', 'nvarchar(max)'),''),

'胸片评分' = [dbo].[EXT_ISNULL](xmlIsland.query(N'//NewCtrl[@Id="胸片评分

"]/Content_Text[last()]').value('(/)[1]',

'nvarchar(max)'),xmlIsland.query(N'//NewCtrl[@Id="胸片评分

1"]/Content_Text[last()]').value('(/)[1]', 'nvarchar(max)'),''),

'CXR 描述' = [dbo].[EXT_ISNULL](xmlIsland.query(N'//NewCtrl[@Id="CXR 描述

"]/Content_Text[last()]').value('(/)[1]',

'nvarchar(max)'),xmlIsland.query(N'//NewCtrl[@Id="CXR 描述

1"]/Content_Text[last()]').value('(/)[1]', 'nvarchar(max)'),''),

'CT 征象描述' = [dbo].[EXT_ISNULL](xmlIsland.query(N'//NewCtrl[@Id="CT 征象描

述"]/Content_Text[last()]').value('(/)[1]',

'nvarchar(max)'),xmlIsland.query(N'//NewCtrl[@Id="CT 征象描述

```
1"]/Content_Text[last()]').value('(/)[1]', 'nvarchar(max)'),'')

'肺炎评分' = [dbo].[EXT_ISNULL](xmlIsland.query(N'//NewCtrl[@Id="肺炎评分

"]/Content_Text[last()]').value('(/)[1]',

'nvarchar(max)'),xmlIsland.query(N'//NewCtrl[@Id="肺炎评分

1"]/Content_Text[last()]').value('(/)[1]', 'nvarchar(max)'),'')

from emr_doc(nolock) inner JOIN PatientBasicInfo(nolock) p on emr_doc.CureNo =

p.VisitNumber

where isDeleted = 0 AND DepartmentCode = '12001020' AND create_date > '2020-05-01'

AND create_date < '2021-05-01' AND template_id = '0AEFB1DD-9ABE-4521-8EB0-

5DDE56598E73'

go
```
